# Beyond Sea Turtles: *Fusarium keratoplasticum* in Eggshells of *Podocnemis unifilis*, a Threatened Amazonian Freshwater Turtle

**DOI:** 10.3390/jof7090742

**Published:** 2021-09-09

**Authors:** Joaquina M. García-Martín, Jullie M. Sarmiento-Ramírez, Javier Diéguez-Uribeondo

**Affiliations:** Departamento de Micología, Real Jardín Botánico-CSIC, 28014 Madrid, Spain; kina@rjb.csic.es (J.M.G.-M.); sugarpie_1co@hotmail.com (J.M.S.-R.)

**Keywords:** bacteria, chelonians, conservation, endangered species, fungi, FSSC, pathogens, Yasuní National Park, yellow-spotted river turtle

## Abstract

The endangered yellow-spotted river turtle (*Podocnemis unifilis*) has experienced a dramatic population decline in the Ecuadorian Amazonia, mainly due to overexploitation of its eggs. To reverse this trend, the Wildlife Conservation Society has developed a head-start program in Yasuní National Park since 2008, but the potential risk that microbes associated with its eggs might represent for hatching success has not been evaluated yet. Members of the *Fusarium* *solani* species complex (FSSC) are involved in egg failure in sea turtles under natural and hatchery conditions, but their role in infecting the eggs of *P. unifilis* is unknown. In this study, we collected eggshells of *P. unifilis* and obtained 50 fungal and bacterial isolates. Some potentially pathogenic fungi of the genera *Fusarium*, *Penicillium* and *Rhizopus* were identified based on molecular data. Most importantly, the sea turtle pathogenic species *F. keratoplasticum* not only was present, but it was the most frequently found. Conversely, we have also isolated other microorganisms, such as *Pseudomonas* or *Phoma*-like species, producing a wide spectrum of antifungal compounds that may have a protective role against fungal diseases. Our survey provides useful information on potential pathogens found in *P. unifilis* eggshells, upon which the success of conservation programs may depend.

## 1. Introduction

Turtles (class Reptilia, order Testudines) are some of the most endangered vertebrates in the world. Indeed, according to International Union for the Conservation of Nature (IUCN) Red List of Threatened Species, most species of this order are threatened, with only 18% being assigned the “least concern” category. The yellow-spotted river turtle (*Podocnemis unifilis* Troschel, 1848; fam. Podocnemididae), native to several South American countries, is currently classified as vulnerable [1], and listed in Appendix II of the Convention on International Trade in Endangered Species of Wild Fauna and Flora [2].

In the Ecuadorian Amazonia, *P. unifilis* inhabits the northwest region of the Yasuní National Park (YNP), where it has experienced a dramatic population decline mainly due to the overexploitation of its eggs for human consumption. Besides, the sale of turtle meat at local illegal markets and the collection of juveniles for pet trade also have a negative influence [3,4,5,6,7]. In order to reverse this trend towards local extinction, the Wildlife Conservation Society (WCS) established a conservation program for *P. unifilis*, commonly referred to as “taricaya” or “charapa”, back in 2008. This ongoing project, self-managed by Kichwa and Waorani Indigenous communities, consists of transplanting nests threatened by egg poachers and floods to hatcheries, and rearing hatchlings in captivity during the first year of life, when mortality reaches a maximum [8,9,10,11].

So far, this collaborative effort has allowed the release of thousands of juveniles into the wild, in exchange for sustainable harvest of eggs and economic alternatives to reduce communities’ dependence on such a resource [9]. However, the real outcome of this, and any other long-term conservation program [12,13,14,15], depends on controlling potential threats that could compromise hatchlings’ survival. To this regard, incubating egg clutches in hatcheries and rearing juveniles in captivity, which involve high densities of eggs and individuals, respectively, could potentially lead to fungal, bacterial, viral and protozoan disease outbreaks [16,17,18,19,20,21].

Previous studies have revealed a broad variety of potentially pathogenic bacteria on sea turtle eggs [22,23,24,25,26]. Moreover, a detailed metagenomic analysis on the microbial community of the eggs of the hawksbill turtle, *Eretmochelys imbricata*, confirmed the presence of some potentially pathogenic bacteria, but also several strains with antifungal activity [27]. In addition, it has been shown that ubiquitous soil fungi belonging to the so-called *Fusarium solani* species complex (hereafter, FSSC), such as *Fusarium falciforme* and *Fusarium keratoplasticum*, can seriously affect hatching success [28]. These species are involved in “sea turtle egg fusariosis” (STEF), an emergent fungal disease linked to egg mortality in endangered sea turtle nests worldwide [29]. Thus, they could compromise the performance of conservation practices based on ex situ incubations.

As for the freshwater turtle *P. unifilis*, several pathogens, such as parasitic nematodes [30,31,32,33], protozoans [34,35], yeasts [36] and bacteria [35,37,38], are known to affect adults. However, little is known about potentially pathogenic or beneficial microorganisms associated with their eggs. In this study we aimed to (1) confirm the presence of *Fusarium* spp. in eggs of *P. unifilis* showing symptoms of *Fusarium* infection, (2) determine the presence of additional fungal and bacterial species, and (3) identify those microorganisms that could either represent a risk or play a protective role, during embryonic development.

## 2. Materials and Methods

### 2.1. Sample Collection

**Figure 1:**
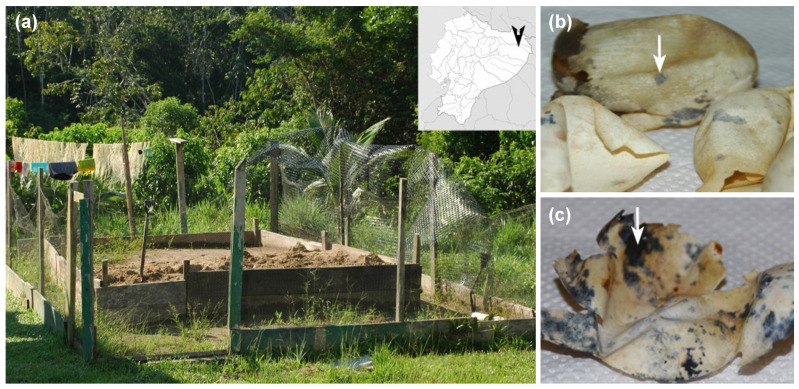
Sampling location at Yasuní National Park (Ecuador) and eggshells of *P. unifilis* with macroscopic signs of *Fusarium* infection (colored spots). The hatcheries were located in the territories of Guiyero, Nueva Providencia and Sani Isla Communities (distributed along the Napo and Tiputini rivers). (**a**) Artificial hatchery located at Guiyero; (**b**) eggshells showing early symptoms of fusariosis (indicated with an arrow); (**c**) eggshells with advanced symptoms (arrow).

We collected 17 eggshells of *P. unifilis* from several nests relocated in three different hatcheries (Figure 1a), at the end of 2011 nesting season. After egg eclosion, eggshells showing typical symptoms of *Fusarium* infection (Figure 1b,c) were manipulated using sterile gloves and placed individually in plastic sealed bags. Samples were maintained in an ice box at 4 °C and transported to the laboratory for processing.

### 2.2. Fungal Isolation and DNA Extraction

Several fragments (ca. 0.5 cm^2^) with obvious signs of infection were excised from each eggshell using a sterile scalpel and plated on peptone glucose agar (PGA) supplemented with ampicillin (100 mg/L). We sub-cultured the resulting fungal colonies to obtain pure cultures, inoculated on potato dextrose agar (PDA) slants in 15 mL tubes. Anexic cultures were incubated at 25 °C for 24 to 72 h, and then permanently stored at 4 °C in the culture collection of the Real Jardín Botánico-CSIC (Madrid, Spain), (Table S1). Total genomic DNA was extracted from pure cultures using a DNeasy Plant Mini Kit (Qiagen, Germantown, MD, USA), according to the manufacturer’s instructions.

### 2.3. PCR Amplification and BLAST-Based Identification of Fungal Isolates

For molecular identification of fungal isolates, we obtained sequences of the nuclear ribosomal ITS region (ITS1, 5.8S and ITS2), using universal primers (Table 1). PCR reactions were performed using Ready-To-Go PCR Beads™ (GE Healthcare Life Sciences, Little Chalfont, UK). Samples were subjected to an initial denaturation step at 94 °C for 5 min, to ensure complete denaturation of the DNA template, as recommended by the manufacturer. After that, the PCR cycling protocol included 5 cycles at 94 °C for 30 s, 54 °C for 30 s and 72 °C for 1 min, followed by 33 cycles at 94 °C for 30 s, 48 °C for 30 s and 72 °C for 1 min, with a final extension step at 72 °C for 10 min [39]. Amplicons were separated by electrophoresis on 2% agarose gels and visualized with an UV transilluminator. The bands of interest were excised from the gels, purified using a QIAquick gel extraction kit (Qiagen, Germantown, MD, USA), and sequenced in both directions by Macrogen (Seoul, Korea) with the same primers used for amplification. Raw sequence edition and consensus assembly were done in Geneious v. 7.1.9 [40]. Sequences were trimmed to exclude low quality and primer-binding sites.

In an attempt to improve the resolution provided by a single molecular region (ITS), we obtained data for two additional molecular regions, i.e., elongation factor-1 alpha (EF-1α) and the nuclear ribosomal large subunit (LSU), from 17 isolates belonging to the FSSC (see Figure S1). Both regions were amplified by PCR using primers previously designed (Table 1).

Cycling parameters for EF-1α amplification were: initial denaturation at 94 °C for 5 min, followed by 40 cycles of denaturation at 94 °C for 30 s, annealing at 55 °C for 90 s, and extension at 68 °C for 2 min, with a final extension step at 76 °C for 5 min. For LSU, the PCR cycling profile consisted of an initial denaturation step at 94 °C for 5 min, followed by 30 cycles of denaturation at 94 °C for 1 min, annealing at 50 °C for 45 s, extension at 72 °C for 1 min, and a final extension step at 72 °C for 7 min [43].

PCR product purification and sequence edition were done as described above. All 62 new fungal sequences generated in this study were submitted to GenBank (Table 2 and Table S1).

### 2.4. Sequence Alignment and Phylogenetic Analyses of the FSSC Isolates

To better place our 17 FSSC isolates in a phylogenetic context, we selected 130 GenBank specimens representing different *Fusarium* species, for which data of two or all three regions analyzed here were available (Table S2). These included, at least, one specimen from each of the subgroups within the FSSC Clade 3 [46], and 20 specimens designated as type material, used to delineate the species in the resulting clusters (Table S2).

Homologous sequences were automatically aligned with MAFFT v. 7.017 [47] with the E-INS-i algorithm and default settings, as implemented in Geneious. Obvious alignment errors were manually adjusted. All alignments used in this study are available under request.

Both Maximum Likelihood (ML) and Bayesian Inference (BI) analyses were first done separately for each region, using resources available in CIPRES [48]. Specifically, individual ML trees were estimated using IQ-TREE v. 1.6.12 [49]. The best-fit model of nucleotide substitution for each dataset was selected with the integrated version of ModelFinder [50]. It was also used to determine the optimal partitioning scheme for each region (ITS was partitioned into ITS1, 5.8S and ITS2, EF-1α was divided into six partitions corresponding to three introns and each codon position of four exons, and LSU was not partitioned). We used the “complete bootstrap” option with 1000 non-parametric bootstrap replicates to assess nodal bootstrap support (BS).

BI single-gene analyses were carried out using the Metropolis-coupled Markov chain Monte Carlo (MCMCMC) method, as implemented in MrBayes v. 3.2.7 [51]. For each dataset, the best-fit substitution model was estimated using the reversible jumping model choice [52], allowing a gamma distributed rate heterogeneity across sites, and a proportion of invariant sites. In the case of the EF-1α and ITS analyses, we used the partitioning schemes selected by ModelFinder, unlinking model parameters across different partitions. In all cases, we used four independent runs of 100 million generations, each with six chains. Trees were sampled every 1000 generations, with the first 25% discarded as burn-in and the posterior probabilities (PP) being calculated from the remaining ones. The Bayesian analyses automatically stopped when the average standard deviation of split frequencies fell below 0.01. Additionally, we assessed run convergence in Tracer v. 1.7.2 [53] by checking the effective sampling size (ESS) values for all parameters (>200).

Single-gene trees were visualized in FigTree v. 1.4.3 [54]. For each locus, both ML and BI analyses yielded very similar topologies, so only the Bayesian tree, showing PP and BS values, is provided (Figures S2–S4). By comparing these trees, we found an incongruent sample, i.e., 153 FUS, that was removed prior to subsequent concatenated analyses.

We predefined 10 partitions for the concatenated dataset (LSU, ITS1, 5.8S, ITS2, each codon position of EF-1α and three EF-1α introns). Again, IQ-TREE was used to choose the best partition scheme and substitution models, and to estimate the concatenated ML tree. The combined Bayesian analysis was performed considering the best partition scheme selected by ModelFinder and the same settings used for the individual analyses. Both ML and BI concatenated trees were visualized and edited in FigTree, and further processed using Adobe Illustrator CS5 (Adobe Systems Inc., San Jose, CA, USA). Only the Bayesian tree showing node supports from both phylogenetic approaches is presented. Members of the FSSC Clade 1 were selected as outgroup, based on a recent phylogenomic study [46].

### 2.5. Bacterial Isolation and DNA Extraction

We used a method previously described for isolating bacterial DNA from other turtle eggs [27], with some modifications. In short, each eggshell fragment was suspended in 10 mL of sterile tap water, vortexed for 2 min and incubated at room temperature for 24 h. Suspensions were diluted (1:10) and then a volume of 50 µL of each dilution was plated on PGA and incubated at 25 °C for five days. The resulting colonies were repeatedly transferred into fresh plates to obtain pure cultures. We prepared bacterial suspensions for long-term storage by inoculating single colonies in 1 mL of sterile tap water into 2 mL tubes, incubating them at 25 °C for 24 h. After that, we added 1 mL of 80% glycerol to the tubes. All suspensions were deposited at −20 °C in the culture collection of the Real Jardín Botánico-CSIC (Table 3). For bacterial DNA extraction, we collected ca. 2 mg of single colonies in 1.5 µL sterile tubes, and followed the same protocol mentioned above.

### 2.6. PCR Amplification, BLAST-Based Identification of Bacterial Isolates, and 16S Phylogeny

The universal primer pair fD2/rP1 (Table 1) was selected to amplify the 16S rDNA gene using Ready-to-Go PCR Beads™ (GE Healthcare Life Sciences, Little Chalfont, UK). PCR reactions were performed in a final volume of 25 µL containing 23 µL of DNA template and 1 µL of each primer (10 µM). The cycling parameters were: initial denaturalization step at 94 °C for 5 min; 35 cycles at 94 °C for 30 s, 52 °C for 30 s and 72 °C for 1 min; and a final extension step at 72 °C for 7 min.

Amplicon purification, sequencing and sequence edition were carried out as previously described. All bacterial 16S sequences newly generated were submitted to GenBank (Table 3).

To roughly identify the bacterial strains isolated here, we compared our edited 16S sequences to GenBank database (Table S3). To establish their phylogenetic relationships and better determine their identity, we conducted phylogenetic analyses as previously described. A set of 137 GenBank 16S sequences from related bacteria, including the type of several genera, was used in these analyses (Table S4). Taking into account that our isolates belong to different phyla and that high levels of sequence divergence between ingroup and outgroup can potentially lead to odd topologies [55,56], we rooted the tree at the mid-point of its longest path, instead of including an outgroup.

## 3. Results

### 3.1. Fungal Isolation and Phylogenetic Analyses of the FSSC Isolates

We obtained 28 fungal isolates from the 17 eggshells of *P. unifilis* analyzed here. According to BLAST searches, 20 of these isolates corresponded to the genus *Fusarium* (BLAST similarity values > 99.5%; Table S1).

To determine whether these isolates belonged to the sea turtle pathogenic FSSC, we run a preliminary phylogenetic analysis based on ITS data (Figure S1). This included most *Fusarium* complexes, and members of the genus *Neonectria* as outgroup according to previous results [46]. Most of the *Fusarium* strains isolated here (17 out of 20) nested within the FSSC, with only three isolates forming part of other major groups, i.e., *Fusarium oxysporum* species complex (FOSC) and, most probably, *Fusarium fujikuroi* species complex (FFSC).

To further investigate their phylogenetic relationships, two other molecular regions were analyzed. Individually, no region was able to fully resolve all relationships within the FSSC, as evidenced by the presence of several polytomies and the generalized low support values recovered for most branches in our single-gene trees (Figures S2–S4).

The isolate 153 FUS appeared sister with full support to the reference sequence of *F. crassum* and one unidentified species in the ITS tree (Figure S2). However, in the EF-1α tree, it nested within a moderately supported clade including the type material of *F. keratoplasticum* and many other samples of this species (Figure S4). Therefore, 153 FUS was removed from the data matrices prior to analyzing them together.

**Figure 2:**
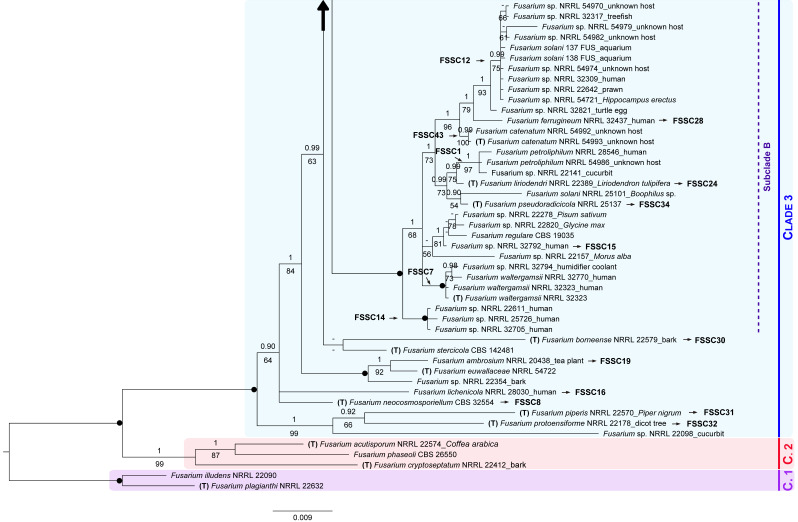

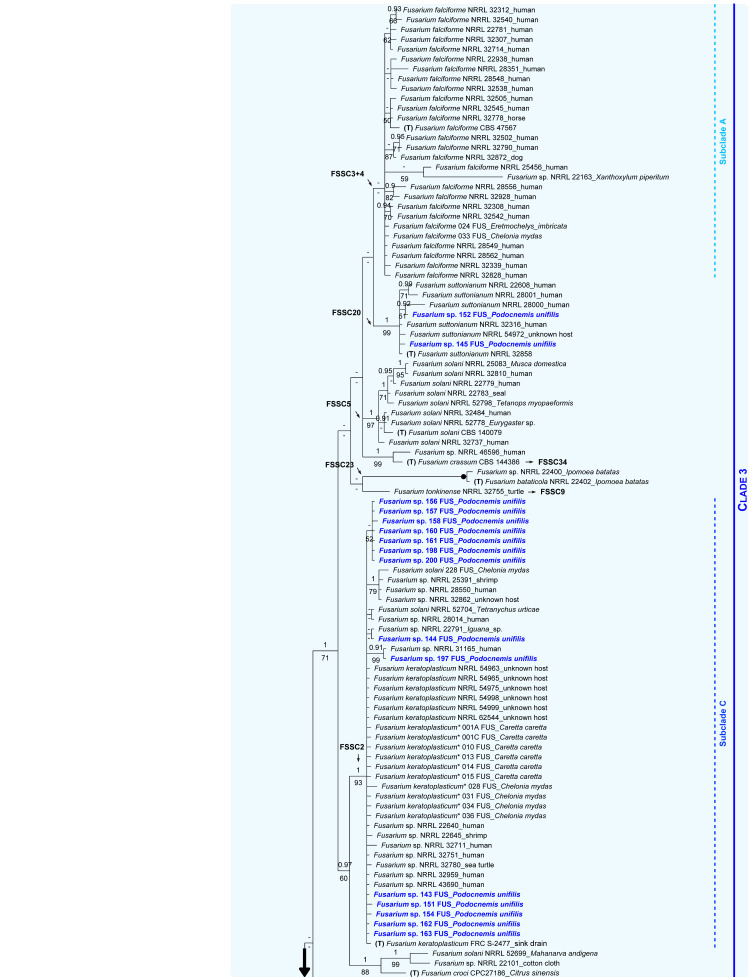
Combined Bayesian tree of the *Fusarium solani* species complex Clade 3, based on 1732 nucleotide positions from 146 isolates, with two members of the FSSC Clade 1 as outgroup. For each isolate, the species name is followed by the corresponding herbarium code, and host if available. Specimens corresponding to *F. keratoplasticum* initially identified as *F. solani* are marked with an asterisk. GenBank specimens corresponding to type material are marked with a “T”. Isolates for which we obtained sequences are in blue bold (153 FUS is not included in this concatenated tree). Clades 1, 2 and 3 represent designations previously proposed by O’Donnell [57]. Subclades A, B and C follow Sarmiento-Ramírez et al. [28]. Different lineages within the Clade 3 follow the nomenclature by O’Donnell et al. [58]. Numbers above and below the branches correspond to Bayesian posterior probability (PP) and maximum likelihood bootstrap values (BS), respectively (shown if PP ≥ 0.90 and BS ≥ 50%). Black solid dots indicate full support in both analyses. The scale bar represents the average number of substitutions per site. A large vertical black arrow indicates that the tree continues along the corresponding branch.

The resulting combined dataset consisted of 1732 characters. Only 277 were parsimony informative given the scarcity of informative characters in each individual data matrix. Out of the 146 isolates analyzed, 144 unique three-locus haplotypes were identified. The corresponding phylogenetic reconstruction (Figure 2) was neither totally resolved. Still, three main monophyletic groups were recovered, all with high support: FSSC Clade 1 (PP = 1, BS = 100%), Clade 2 (PP = 1, BS = 99%), and Clade 3 (PP = 1, BS = 100%).

Within the FSSC Clade 3, some specimens occupied an undefined phylogenetic position. Besides, several minor clades and three recognizable large monophyletic groups were recovered, i.e., Subclade A (PP = 0.79, BS = 33%), Subclade B (PP = 1, BS = 100%), and Subclade C (PP = 1, BS = 93%).

All 17 FSSC isolates were linked to a species reference sequence (i.e., generated from type material). Specifically, 14 isolates clustered in the Subclade C along with the type strain *F. keratoplasticum* FRC S-2477 and additional specimens of this species. Sister to the Subclade A with no support (PP = 0.70, BS = 14%), we found a clade formed by 145 FUS, 152 FUS, *F. suttonianum* NRRL 32858 (type material), and other representatives of this species (PP = 1, BS = 99%).

The remaining internal relationships within the FSSC Clade 3 are not detailed for the sake of brevity.

Considering the phylogenetic relationships among the isolates of the FSSC Clade 3 and those corresponding to type material (retrieved from GenBank) in this multigene phylogeny, but also in the ITS tree (Figure S1), most isolates were identified as *F. keratoplasticum* (Table 2).

**Table 2:** FSSC Clade 3 isolates obtained from the eggshells of *P. unifilis* analyzed in this study.

Isolate	Geographic Origin ^a^	Species ID ^b^	GenBank Accession No.		
			ITS	LSU	EF-1α
143 FUS	Guiyero	*F. keratoplasticum*	MW390926	MW390975	MW389342
144 FUS	Guiyero	*F. keratoplasticum*	MW390927	MW390976	MW389343
145 FUS	Guiyero	*F. suttonianum*	MW390928	MW390977	MW389356
151 FUS	Guiyero	*F. keratoplasticum*	MW390930	MW390978	MW389344
152 FUS	Nueva Providencia	*F. suttonianum*	MW390931	MW390979	MW389357
153 FUS	Sani Isla	*F.* cf. *crassum*	MW390932	MW390980	MW389341
154 FUS	Sani Isla	*F. keratoplasticum*	MW390933	MW390981	MW389345
156 FUS	Sani Isla	*F. keratoplasticum*	MW390935	MW390982	MW389346
157 FUS	Sani Isla	*F. keratoplasticum*	MW390936	MW390983	MW389347
158 FUS	Undetermined	*F. keratoplasticum*	MW390937	MW390984	MW389348
160 FUS	Undetermined	*F. keratoplasticum*	MW390939	MW390985	MW389349
161 FUS	Undetermined	*F. keratoplasticum*	MW390940	MW390986	MW389350
162 FUS	Undetermined	*F. keratoplasticum*	MW390941	MW390987	MW389351
163 FUS	Undetermined	*F. keratoplasticum*	MW390942	MW390988	MW389352
197 FUS	Guiyero	*F. keratoplasticum*	MW390949	MW390989	MW389353
198 FUS	Sani Isla	*F. keratoplasticum*	MW390950	MW390990	MW389354
200 FUS	Sani Isla	*F. keratoplasticum*	MW390952	MW390991	MW389355

^a^ Guiyero: 00°36′14″ S, 76°28′03” W; Nueva Providencia: 00°29′35′′ S, 76°28′21″ W; Sani Isla: 00°28′30″ S, 76°18′37′′ W. ^b^ Species identification based on the multigene phylogenetic tree presented in this study.

### 3.2. Bacterial Isolates and Phylogenetic Analyses

**Figure 3:**
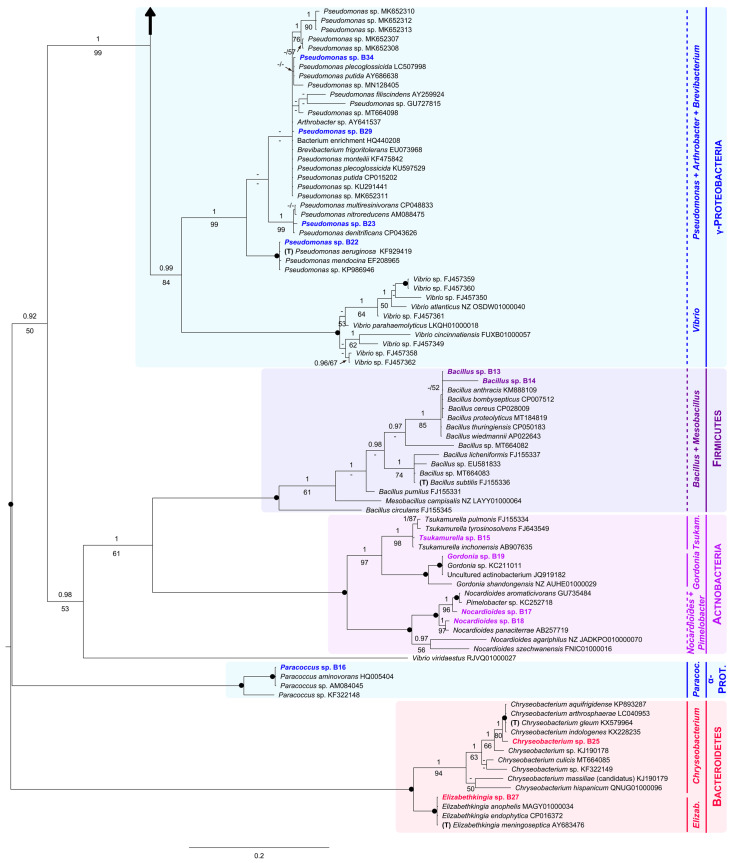

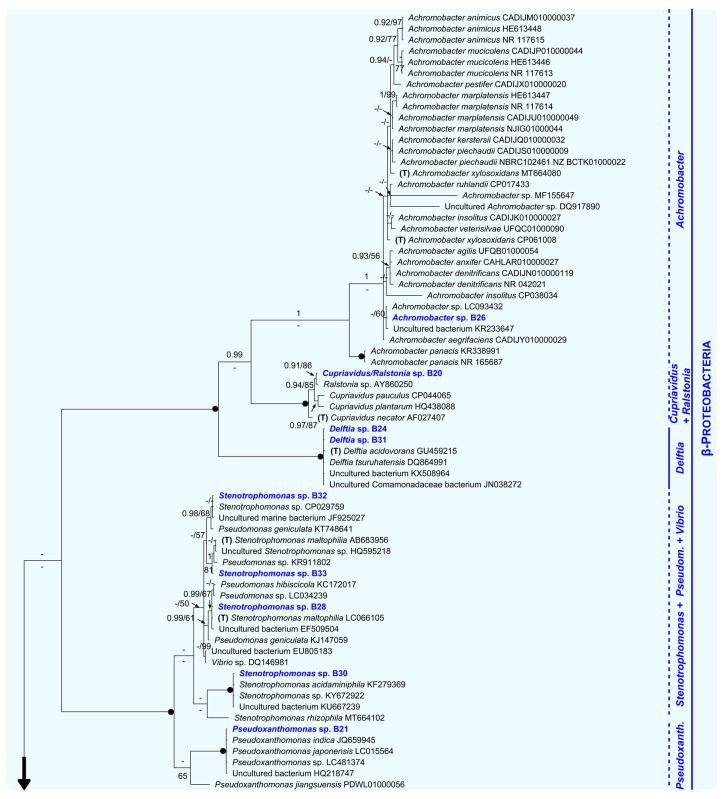
Unrooted Bayesian tree of the bacteria isolated from eggshells of *P. unifilis*, and allies. For each isolate, the species name is followed by the GenBank accession number. Sequences obtained in this study are in bold. Specimens representing the type of a genus are marked with a “T”. Numbers above and below the branches correspond to Bayesian posterior probability (PP) and maximum likelihood bootstrap values (BS), respectively (shown if PP ≥ 0.90 and BS ≥ 50%). Black solid dots indicate full support in both analyses. The scale bar represents the average number of substitutions per site. Discontinuous vertical lines indicate either unsupported or non-monophyletic genera. Vertical lines correspond to well-supported monophyletic genera. A large vertical black arrow indicates that the tree continues along the corresponding branch.

We obtained 22 bacterial isolates and analyzed them in a phylogenetic context (Figure 3). Specifically, in our 16S phylogenetic tree, six main clades were recovered: (1) subphylum Beta-Proteobacteria, (2) subphylum Gamma-Proteobacteria, (3) phylum Firmicutes, (4) phylum Actinobacteria, (5) subphylum Alfa-Proteobacteria, and (6) phylum Bacteroidetes (Figure 3).

Within the Beta-Proteobacteria clade, the isolate B26 and all representatives of the genus *Achromobacter* analyzed here, including the type (*A. xylosoxidans*), constituted a group only supported in the Bayesian analysis (PP = 1, BS = 33%). A fully supported multispecies group (PP = 1, BS = 100%) comprised the isolate B20, the type of the genus *Cupriavidus*, and one member of *Ralstonia*. Sister with full support to both mentioned assemblages, we found another highly supported monophyletic group (PP = 1, BS = 100%). This was formed by B24, B31, two species of the genus *Delftia* (including the type, *D. acidovorans*), and two unidentified specimens. Additionally, within Beta-Proteobacteria, B28, B30, B32 and B33 formed an unsupported group with several species of the genus *Pseudomonas*, a single representative of *Vibrio*, and the type of *Stenotrophomonas* (*S. maltophilia*, recovered as polyphyletic). Closely related to this unsupported group, we found a small robust clade constituted by the isolate B21 and several species of *Pseudoxanthomonas* (PP = 1, BS = 100%).

Within the Gamma-Proteobacteria clade (PP = 0.99, BS = 84%), B22, B23, B29 and B34 nested within a strongly supported group (PP = 1, BS = 99%) that comprised most *Pseudomonas* analyzed here (including the type, *P. aeruginosa*), and two representatives of the genera *Arthrobacter* and *Brevibacterium*.

As for the Firmicutes clade (PP = 1, BS = 100%), it contained both isolates B13 and B14, all sequences of *Bacillus* analyzed here, including that of the type (*B. subtilis*), and one representative of *Mesobacillus.*

Sister to Firmicutes, with moderate support (PP = 1, BS = 61%), we found the Actinobacteria as monophyletic, receiving full support. It was further divided into three groups: (1) the well-supported *Tsukamurella* clade (PP = 1, BS = 98%), including the isolate B15; (2) the *Gordonia* clade (PP = 1, BS = 100%), holding the isolate B19; and (3) the *Nocardiodes*/*Pimelobacter* clade, also fully supported (PP = 1, BS = 100%), harboring B17 and B18.

The Alfa-Proteobacteria clade (PP = 1, BS = 100%) appeared in a distant position to both subphyla Gamma and Beta-Proteobacteria. It was formed by the isolate B16 and three members of the genus *Paracoccus*.

Finally, within the Bacteroidetes clade (PP = 1, BS = 100%), the isolates B25 and B27 were part of two robust clades constituted by members of the genera *Chryseobacterium* (PP = 1, BS = 94%) and *Elizabethkingia* (PP = 1, BS = 100%), respectively.

To summarize, the bacteria isolated from the eggshells of *P. unifilis* corresponded to 13 different genera belonging to four phyla (Table 3).

**Table 3:** Bacterial isolates obtained from the eggshells of *P. unifilis* analyzed in this study.

Isolate	Geographic Origin	Genus ID ^a^	Phylum	16S GenBank Accession No.
B13	Nueva Providencia	*Bacillus*	Firmicutes	MW391108
B14	Nueva Providencia	*Bacillus*	Firmicutes	MW391109
B15	Nueva Providencia	*Tsukamurella*	Actinobacteria	MW391110
B16	Guiyero	*Paracoccus*	Proteobacteria	MW391111
B17	Guiyero	*Nocardioides*	Actinobacteria	MW391112
B18	Sani Isla	*Nocardioides*	Actinobacteria	MW391113
B19	Sani Isla	*Gordonia*	Actinobacteria	MW391114
B20	Sani Isla	*Cupriavidus* or *Ralstonia*	Proteobacteria	MW391115
B21	Sani Isla	*Pseudoxanthomonas*	Proteobacteria	MW391116
B22	Sani Isla	*Pseudomonas*	Proteobacteria	MW391117
B23	Sani Isla	*Pseudomonas*	Proteobacteria	MW391118
B24	Sani Isla	*Delftia*	Proteobacteria	MW391119
B25	Undetermined	*Chryseobacterium*	Bacteroidetes	MW391120
B26	Undetermined	*Achromobacter*	Proteobacteria	MW391121
B27	Undetermined	*Elizabethkingia*	Bacteroidetes	MW391122
B28	Undetermined	*Stenotrophomonas*	Proteobacteria	MW391123
B29	Undetermined	*Pseudomonas*	Proteobacteria	MW391124
B30	Undetermined	*Stenotrophomonas*	Proteobacteria	MW391125
B31	Undetermined	*Delftia*	Proteobacteria	MW391126
B32	Undetermined	*Stenotrophomonas*	Proteobacteria	MW391127
B33	Undetermined	*Stenotrophomonas*	Proteobacteria	MW391128
B34	Undetermined	*Pseudomonas*	Proteobacteria	MW391129

^a^ Genus identification based on the 16S phylogenetic tree presented in this study.

## 4. Discussion

The yellow-spotted river turtle, *Podocnemis unifilis*, is one of the most threatened reptiles in the Ecuadorian Amazonia. This has made it necessary to implement conservation actions through a collaborative effort among WCS and several Indigenous communities historically linked to the YNP. Specifically, the head start program developed by WCS has focused its strategy on egg translocations and their incubation in protected hatcheries. However, so far, the presence of microorganisms representing a potential risk for hatching success has not been evaluated under hatchery conditions.

Here, we describe, for the first time, the fungal and microbial communities associated with eggs of *P. unifilis* apparently colonized by *Fusarium* spp. Given the difficulties to collect samples from threatened species, a limited number of eggshells could be analyzed. Therefore, our results provide a glimpse into the fungi and bacteria associated with its eggs.

The multigene tree shown in Figure 2 was substantially congruent with that presented in a recent phylogenomic study [46]. Based on our tree, several fungi isolated from the eggshells of *P. unifilis* were revealed to be members of the FSSC Clade 3. Specifically, most isolates corresponded to *F. keratoplasticum* (lineage FSSC 2), a soil-borne species globally distributed. Notably, previous studies have reported that this and other species of the FSSC constitute a real threat to sea turtle nests worldwide, especially to those subject to environmental stressors, among others, inundation and clay/silt composition of nests [28,59,60,61].

Under natural conditions, the nests of *P. unifilis* are often exposed to flooding, which might favor the development of *Fusarium* in the eggs. Besides, clutches incubated under hatchery conditions are defenseless against additional stressors leading to the accumulation of pathogen spores and the spread of contaminants (e.g., manipulation, high density of nests, and reusing the substrate and the same wood frame for several seasons). These factors might exacerbate *Fusarium* development and eggs contamination by this and other microorganisms that could seriously affect hatching success [62,63].

While we cannot conclude whether *F. keratoplasticum* directly contributes to *P. unifilis* embryo death, this species has been not only isolated from unhatched loggerhead eggs, but it has been proved responsible for sea turtle mass mortalities related to STEF [27,28,60,61,64]. Thus, research efforts should focus on further characterizing this fungus and its potential pathogenicity in *P. unifilis*. Likewise, considering that *F. keratoplasticum* is less frequently isolated in in situ than in relocated sea turtle nests [60,65], it should also be investigated whether common management practices, such as bare-hand contact with the eggs or reusing the hatchery structure, increase the risk of fungal outbreaks for *P. unifilis*.

Apart from causing STEF, *F. keratoplasticum* is one of the most frequent etiological agents of mycotic keratitis, onychomycosis and disseminated infections in immunocompromised persons [66,67,68], who can get infected by inhalation of microconidia and/or skin penetration [69,70]. Considering the potential vulnerability of Ecuadorian Indigenous people, exposed to untreated toxic wastes from oil industries [71,72,73], and in close contact with the eggs, every attempt to prevent *F. keratoplasticum* infection from occurring and spreading should be made. We recommend implementing basic preventive measures, such as using protective masks and single-use gloves, hand washing and disinfection when handling eggs and/or hatchlings.

Although predominant within the FSSC Clade 3, *F. keratoplasticum* was not the only fungal species found in the eggshells of *P. unifilis*. We also recovered three isolates phylogenetically related to *F. suttonianum* (Figure 2) and, possibly, *F. crassum* (Figures S1 and S2). The former is an uncommon human pathogenic species, reported from blood samples, that can cause human keratitis [58], while *F. crassum* has been isolated from numerous hosts, including human clinical samples [74]. However, to the best of our knowledge, none of these species has been previously isolated from turtle eggs.

Additionally, three samples were identified as members of other complexes. More in detail, although ITS data from the type material of *F. oxysporum* could not be included in our analyses, several specimens of this species appeared sister to 199 FUS. For this reason, this isolate is considered here as a member of the FOSC, and it could correspond to *F. oxysporum*, or a closely related species (Figure S1). *Fusarium oxysporum* has been repeatedly found in both nests and failed eggs of several sea turtles [75,76,77,78,79,80]. Additionally, its presence in *Dermochelys coriacea* eggs negatively affects the size of the hatchlings [81], which could have undesirable consequences on their survival. The remaining two *Fusarium* isolates, 168 FUS and 170 FUS, form part of the FFSC, which is recovered as paraphyletic in our preliminary ITS tree by including members of the FOSC and other complexes (Figure S1). The relationships among *F. concentricum*, *F. fujikuroi*, *F. proliferatum* and both isolates are unclear, but they share the same ITS sequence with a specimen of the first mentioned species, while only one base change differentiates their ITS sequence from those obtained from the type material of *F. fujikuroi* and several specimens of *F. proliferatum*. Some members of the FFSC cause severe diseases in economically important plants [82,83,84,85,86]. Moreover, *F. fujikuroi* is known to act as an entomopathogenic fungi but, to our knowledge, only *F. proliferatum* has been previously associated to mycotic infections in turtle eggs [87]. Additional data from more informative loci are needed to firmly establish the identity of this pair of isolates.

Other than *Fusarium* spp., the fungi isolated from our samples also included the species *Rhizopus microsporus*, and one member of the genus *Penicillium*. To our knowledge, *R*. *microsporus* has not been previously reported from reptiles. In contrast, its close relatives *R. stolonifer* and *R. oryzae* have been isolated from eggs and nests of green turtles, *Chelonia mydas* [75,78,88,89]. *Rhizopus stolonifer* has been also found in soft-shell turtles, *Apalone ferox*, affected by cutaneous mycosis [90]. Interestingly, several members of the genus *Rhizopus* produce mycotoxins [75,91] that might be harmful for the turtle embryonic development, although this remains to be proved. As for *Penicillium*, some species of this genus have been identified in nests, eggs and skin lesions of numerous chelonians, including terrapins, tortoises and sea turtles [75,78,89,92,93,94]. They are known for their mycotoxigenic properties, thought to be detrimental for developing eggs under hatchery conditions [95]. Furthermore, some *Penicillium* species have been related to bronchopneumonia in sea turtles [96] and so, caution is recommended to prevent the risk of respiratory tract allergies for people handling both eggs and hatchlings of *P. unifilis*.

Members of the family Didymellaceae (possibly *Allophoma*, *Didymella*, and *Phoma* and allied genera) also formed part of the microflora of *P. unifilis* eggs. These taxa comprise plant pathogenic, saprobic and endophytic species associated with a wide range of hosts, including crops [97,98,99,100]. More interestingly, the species *Phoma multirostrata* has been isolated from eggs of *C. mydas* [64], and several congenerics synthetize antifungal compounds with broad-spectrum activity [101,102]. Hence, it would be interesting to investigate the role of *Phoma* spp. and its relatives on pathogenicity or mitigation of *Fusarium* infections in sea and freshwater turtle eggs.

While this constitutes the first report of fungi associated with eggshells of *P. unifilis*, we also provide novel data on the accompanying bacteria. We could not identify most of them at the species level, but members of the phyla Proteobacteria and Actinobacteria prevailed over other species, as occurs in sea turtle eggs [27]. In particular, the bacterial community associated with the eggs of *P. unifilis* seems to be dominated by Gram-negative aerobic bacteria normally present in the environment, or as part of the turtle microbiota, that may act as opportunistic pathogens under stressful conditions [24,103]. Among such bacteria we found *Pseudomonas* and *Stenotrophomonas*, both previously isolated from chelonians [24,104,105,106]. The genus *Pseudomonas* is known to be part of the normal microbiota of the mouth and cloaca of several turtles [22,25,103,104,105], including *P. unifilis* [37]. Besides, *Pseudomonas* spp. have been also isolated from eggs, and their presence has been linked to low hatching success [22,23,24,25,104]. The only bacterium identified at the species level, *Stenotrophomonas maltophilia*, has been isolated from several diseased adult animals [107,108], and also from unhatched sea turtle eggs [24,106,107]. Notably, infections caused by *Pseudomonas* spp. and *Stenotrophomonas* spp. are especially difficult to control because of their high resistance to most antibiotics [25,58,103,109,110,111,112]. So, apart from being a potential reproductive hazard to *P. unifilis*, they may have an important role on the dissemination of antimicrobial resistance, an increasingly concerning issue, and may also pose a health risk for immunocompromised people [26,105,113]. Therefore, it is recommendable to take certain precautions while handling eggs of *P. unifilis.*

On the other hand, some *Pseudomonas* species contribute to the natural soil suppressiveness against several fungal pathogens, including *Fusarium* [114,115,116]. Thus, the potential role of *Pseudomonas* as a pathogen and/or as an antagonistic of *Fusarium* disease on *P. unifilis* eggs needs investigation.

The second most abundant phylum, Actinobacteria, was represented by *Gordonia*, *Nocardioides* and *Tsukamurella*. Species of these genera have been described as pathogens in snakes [117,118] and tortoises [119]. However, there is no evidence of their presence in turtle eggs. Other bacteria found in *P. unifilis* eggs include *Bacillus* spp. (phylum Firmicutes), well-known human pathogens that probably account for developmental arrest in turtle embryos [26]. We have also identified some members of Bacteroidetes, one of the three most abundant phyla affecting both wild-captured and stranded green turtles [120]. Among them, we found the genus *Elizabethkingia*, which has also been regarded as potentially pathogenic for reptiles [121]. Consequently, in-depth studies are needed to further characterize these strains, which could eventually represent a serious hazard. Despite the limitations derived from the absence of a microbiological assessment, our survey provides useful information on the bacteria found in eggs of *P. unifilis* upon which the success of WCS conservation program depends.

## 5. Conclusions

This is the first molecular study on the microbiota associated to *P. unifilis* eggshells. It significantly contributes to the existing literature on fungal and bacterial contamination of freshwater turtle eggs. Most importantly, this study points to a potential major problem for the conservation of *P. unifilis* that is the extended presence of the pathogenic fungus *F. keratoplasticum* in its eggs. If it were proved that this fungal species causes a disease, then, by analogy with STEF, it could be referred to as “freshwater turtle egg fusariosis” (FTEF). On the other hand, we have also identified other fungi and bacteria that might have antagonistic activity against *Fusarium*. These findings have direct application on the WCS conservation program since feasible measures could be easily implemented to prevent *Fusarium* disease development in eggs of *P. unifilis*, and to protect individuals working in hatcheries. Further studies on the microbiota of *P. unifilis* eggs are necessary for a better understanding of their pathogenic or beneficial effects, and their role on the conservation of the yellow-spotted river turtle.

## Figures and Tables

**Table 1:** Primers used to amplify different molecular regions analyzed in this study.

Organism/ Target Gene	Primer Name ^a^	Primer Sequence (5′→3′)	Reference
**Fungi**			
ITS	ITS5 (f)	GGAAGTAAAAGTCGTAACAAGG	[41]
	ITS4 (r)	TCCTCCGCTTATTGATATGC	
EF-1α	EF-1 (f)	ATGGGTAAGGA(A/G)GACAAGAC	[42]
	EF-2 (r)	GGA(G/A)GTACCAGT(G/C)ATCATGTT	
LSU	LR0R (f)	ACCCGCTGAACTTAAGC	[43,44]
	LR5 (r)	ATCCTGAGGGAAACTTC	
**Bacteria**			
16S	fD2	AGAGTTTGATCATGGCTCAG	[45]
	rP1	ACGGTTACCTTGTTACGACTT	

^a^ If not indicated by the primer name itself, forward and reverse primers are marked with (f) and (r), respectively.

## Data Availability

All data supporting the findings of this study are presented within this article and online Supplementary Materials. Sequences generated here are deposited in GenBank. Any additional data are available on request to the corresponding author.

## Supplementary Materials

The following are available online at https://www.mdpi.com/article/10.3390/jof7090742/s1, Figure S1: Preliminary Bayesian ITS tree of all 20 *Fusarium* isolated from *P. unifilis* eggshells, with *Neonectria* as outgroup, Figure S2: Bayesian ITS tree of FSSC isolates, based on 499 nucleotides, with members of the FSSC Clade 1 as outgroup, Figure S3: Bayesian LSU tree of FSSC isolates, based on 520 nucleotides, with members of the FSSC Clade 1 as outgroup, Figure S4: Bayesian EF-1α tree of FSSC isolates, based on 713 nucleotides, with members of the FSSC Clade 1 as outgroup, Table S1: Fungal isolates obtained from *P. unifilis* eggshells, and putative identity based on ITS data, Table S2: GenBank sequences from 130 fungal specimens used in phylogenetic analyses, Table S3: Bacteria isolated from *P. unifilis* eggshells and putative identity based on 16S Blast results, Table S4: GenBank 16S sequences from related bacterial species used in phylogenetic analyses.
