# Supplementary material for: Beyond Sea Turtles: Fusarium keratoplasticum in Eggshells of Podocnemis unifilis, a Threatened Amazonian Freshwater Turtle
Source: J Fungi (Basel). 2021 Sep 9;7(9):742. doi: 10.3390/jof7090742 (PMC8470610; doi:10.3390/jof7090742)
Supplement: Supplementary file 1 [file jof-07-00742-s001.zip › jof-1352330-supplementary.pdf]

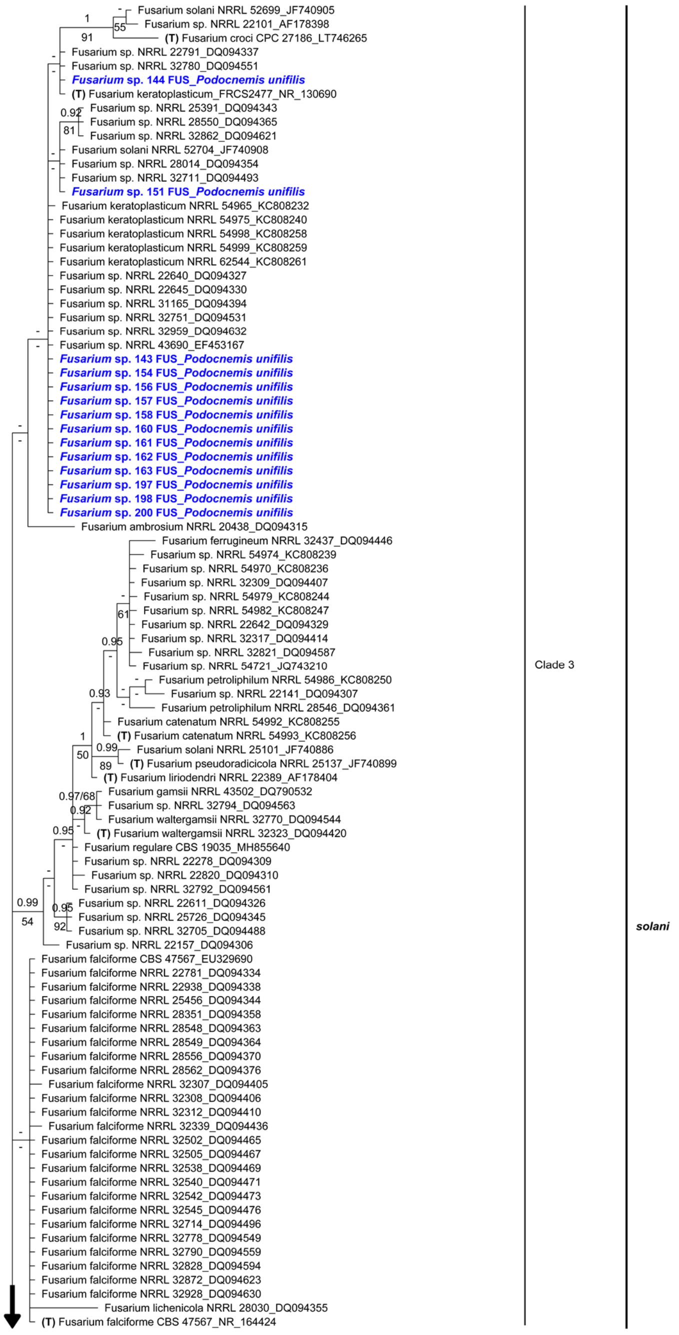

Figure S1. Preliminary Bayesian ITS tree of all 20 *Fusarium* isolated from *P. unifilis* eggshells, with *Neonectria* as outgroup. Species names are followed by herbarium codes and GenBank accession numbers. Sequences obtained here are in bold. Type strains are marked with a "T". An interrupted branch (//) indicates its length has been reduced. Numbers above and below the branches represent Bayesian posterior probability (PP) and maximum likelihood bootstrap values (BS), respectively (shown only if PP ≥ 0.90 and BS ≥ 50%). Solid dots indicate full support in both analyses. Species complexes are indicated in the right side of the figure (lines are discontinuous if recovered as non-monophyletic). The scale bar represents the average number of substitutions per site.

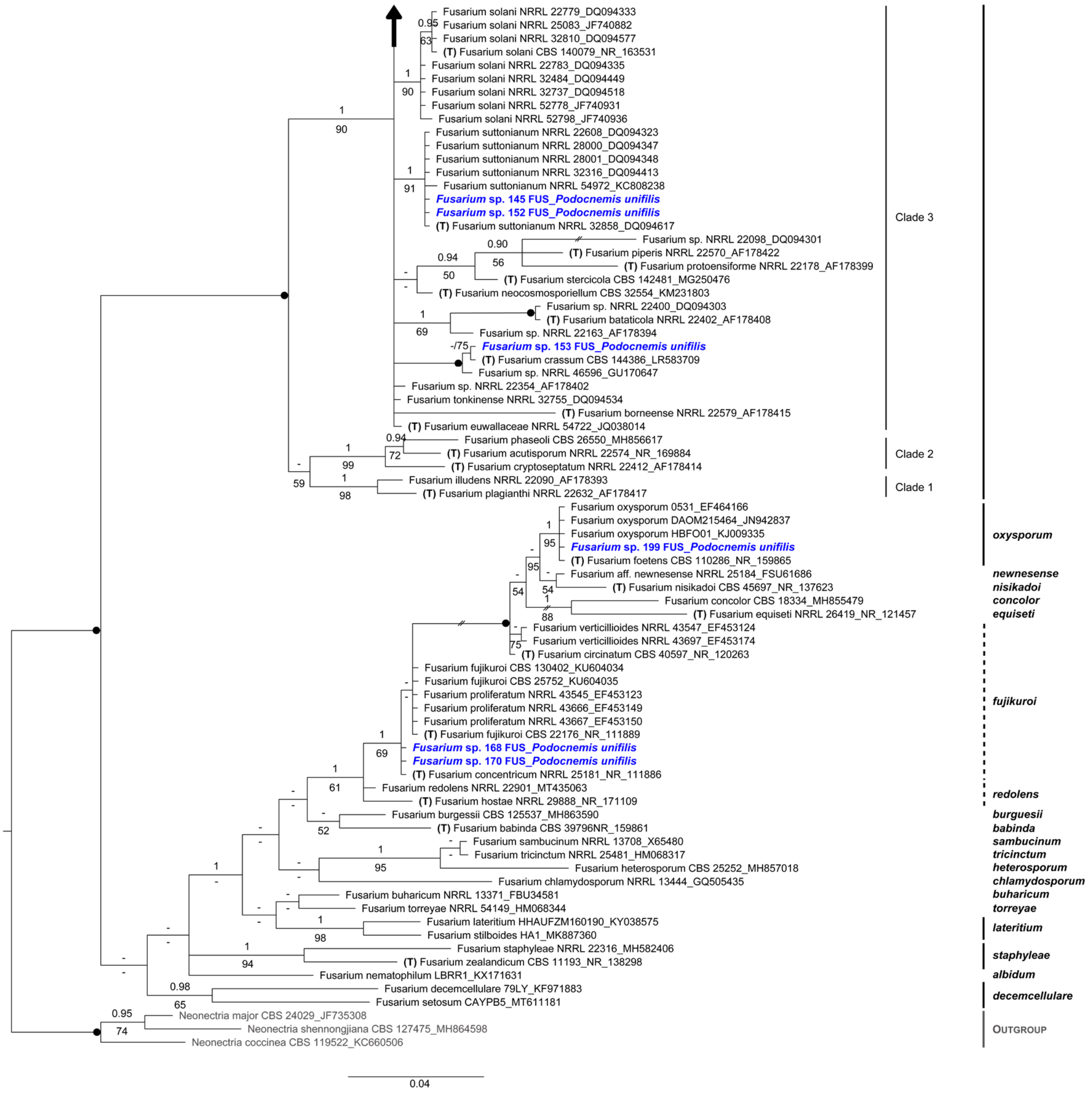

Figure S1. (Continuation).

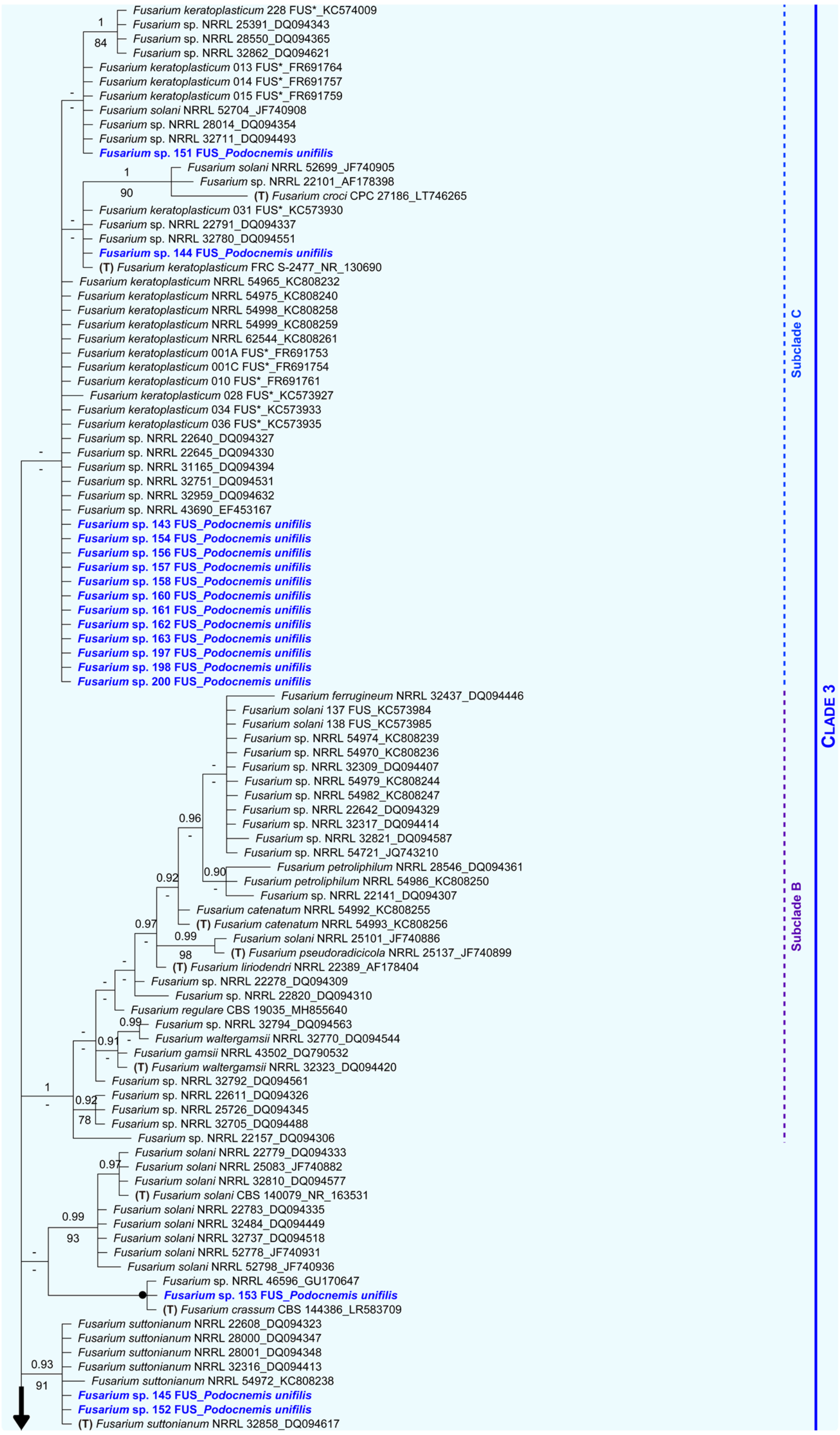

Figure S2. Bayesian ITS tree of FSSC isolates, based on 499 nucleotides, with members of the FSSC Clade 1 as outgroup. Species names are followed by herbarium codes and GenBank accession numbers. Sequences obtained here are in bold. Type strains are marked with a "T". Specimens corresponding to *F. keratoplasticum*, initially identified as *F. solani*, are marked with an asterisk. Clades 1, 2 and 3 represent designations proposed by O'Donnell et al. [57]. Subclades B and C follow Sarmiento-Ramírez et al. [28]. Numbers above and below the branches represent Bayesian posterior probability (PP) and maximum likelihood bootstrap values (BS), respectively (shown only if PP ≥ 0.90 and BS ≥ 50%). Solid dots indicate full support in both analyses. The scale bar represents the average number of substitutions per site.

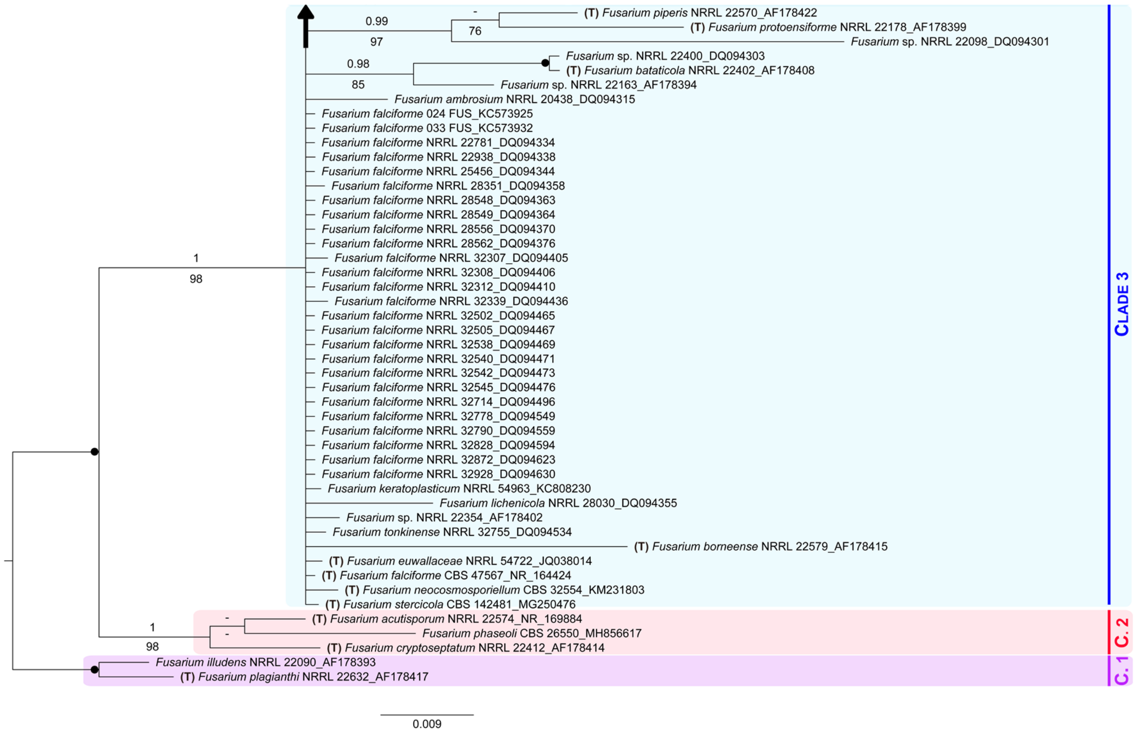

Figure S2. (Continuation).

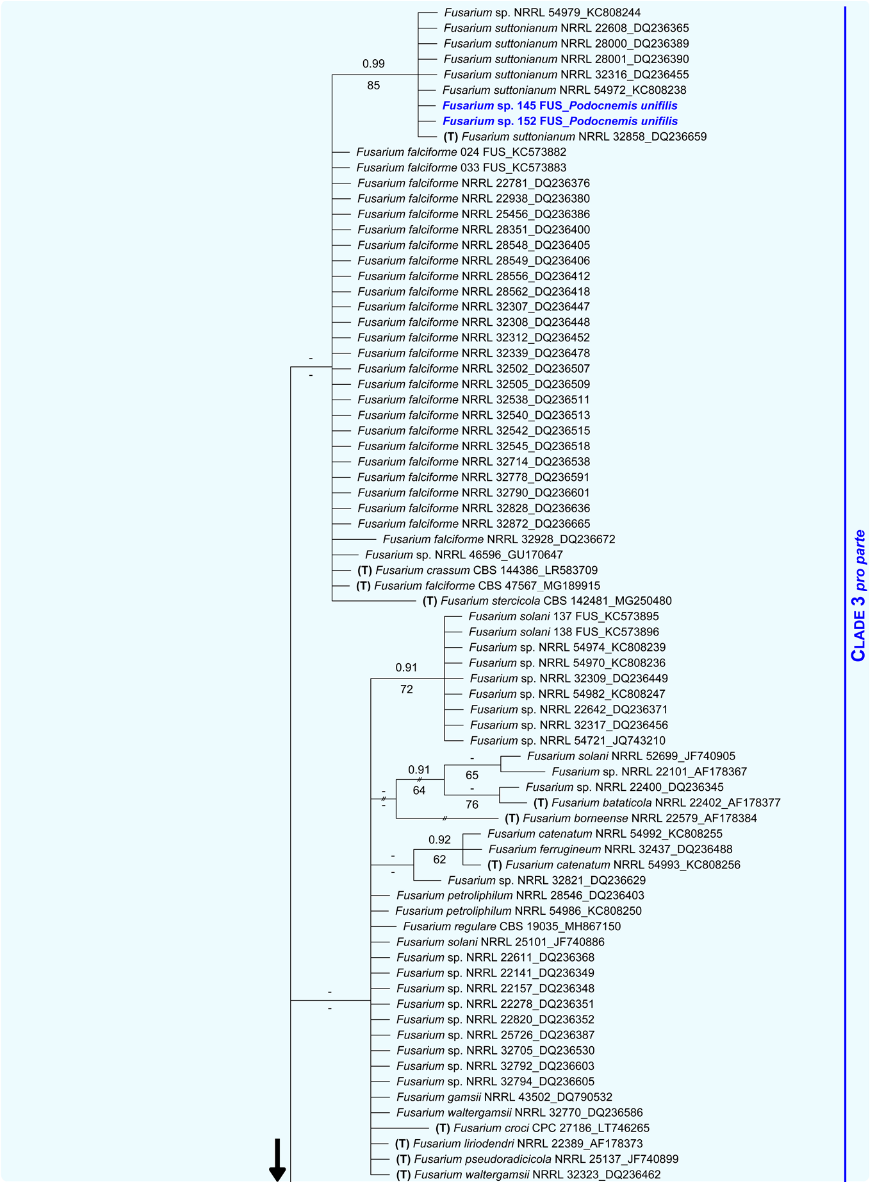

Figure S3. Bayesian LSU tree of FSSC isolates, based on 520 nucleotides, with members of the FSSC Clade 1 as outgroup. Species names are followed by herbarium codes and GenBank accession numbers. Sequences obtained here are in bold. Type strains are marked with a "T". Specimens corresponding to *F. keratoplasticum*, initially identified as *F. solani*, are marked with an asterisk. Clades 1, 2 and 3 represent designations proposed by O'Donnell et al. [57]. Numbers above and below the branches represent Bayesian posterior probability (PP) and maximum likelihood bootstrap values (BS), respectively (shown only if PP ≥ 0.90 and BS ≥ 50%). An interrupted branch (//) indicates its length has been reduced for representation purposes. The scale bar represents the average number of substitutions per site.

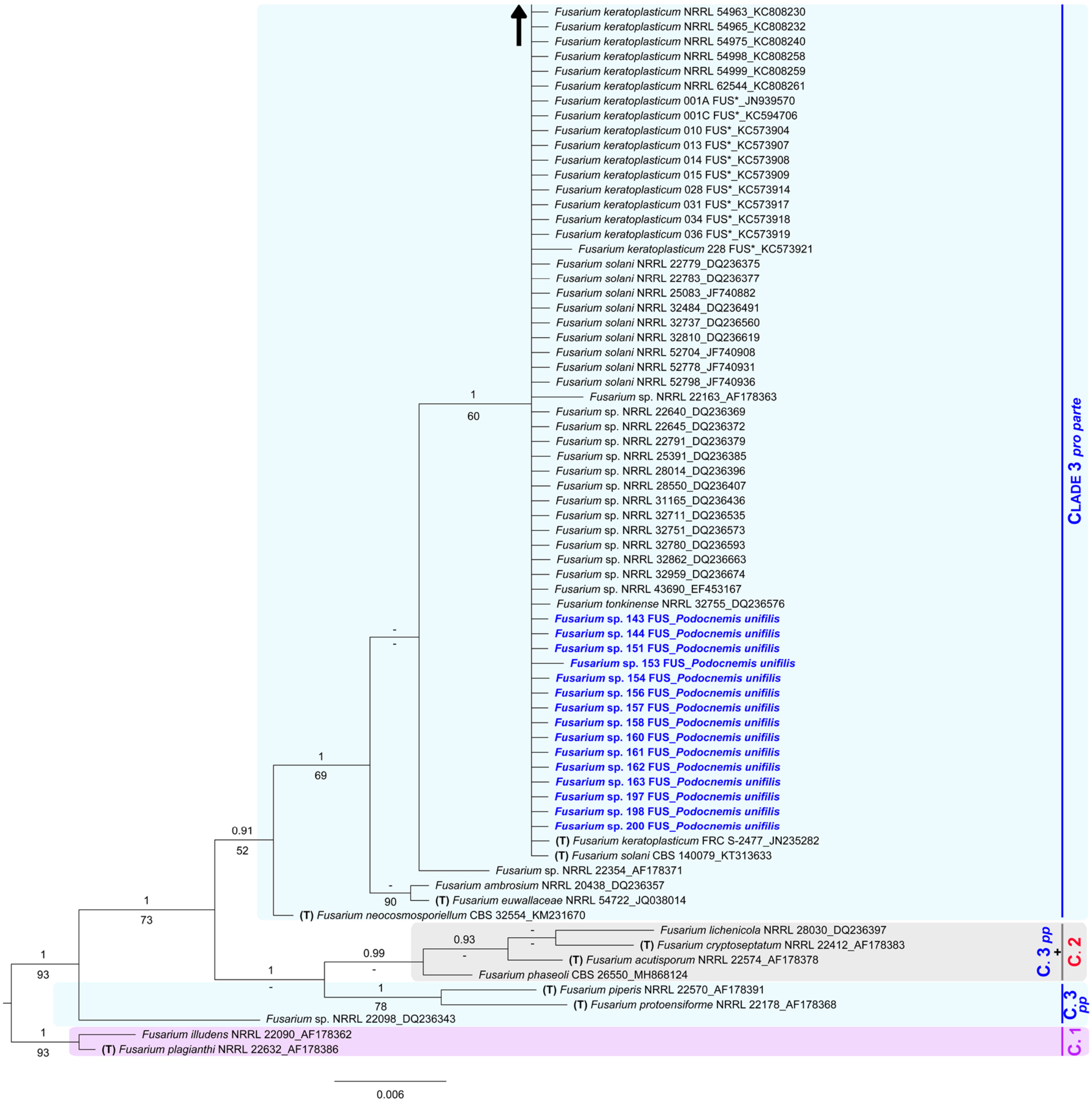

Figure S3. (Continuation).

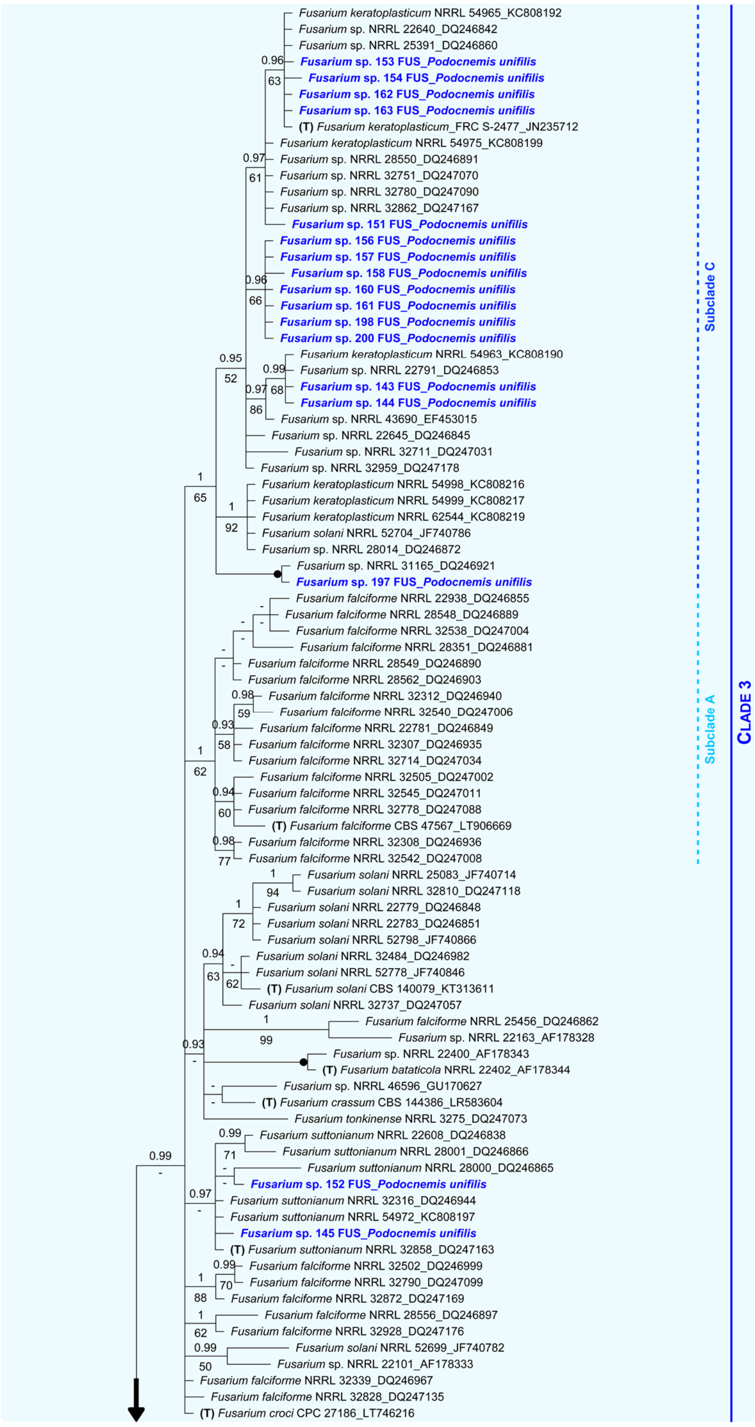

Figure S4. Bayesian EF-1α tree of FSSC isolates, based on 713 nucleotides, with members of the FSSC Clade 1 as outgroup. Species names are followed by herbarium codes and GenBank accession number. Sequences obtained here are in bold. Type strains are marked with a "T". Clades 1, 2 and 3 represent designations proposed by O'Donnell et al. [57]. Subclades A, B and C follow Sarmiento-Ramírez et al. [28]. Numbers above and below the branches represent Bayesian posterior probability (PP) and maximum likelihood bootstrap values (BS), respectively (shown only if PP ≥ 0.90 and BS ≥ 50%). Solid dots indicate full support in both analyses. The scale bar represents the average number of substitutions per site.

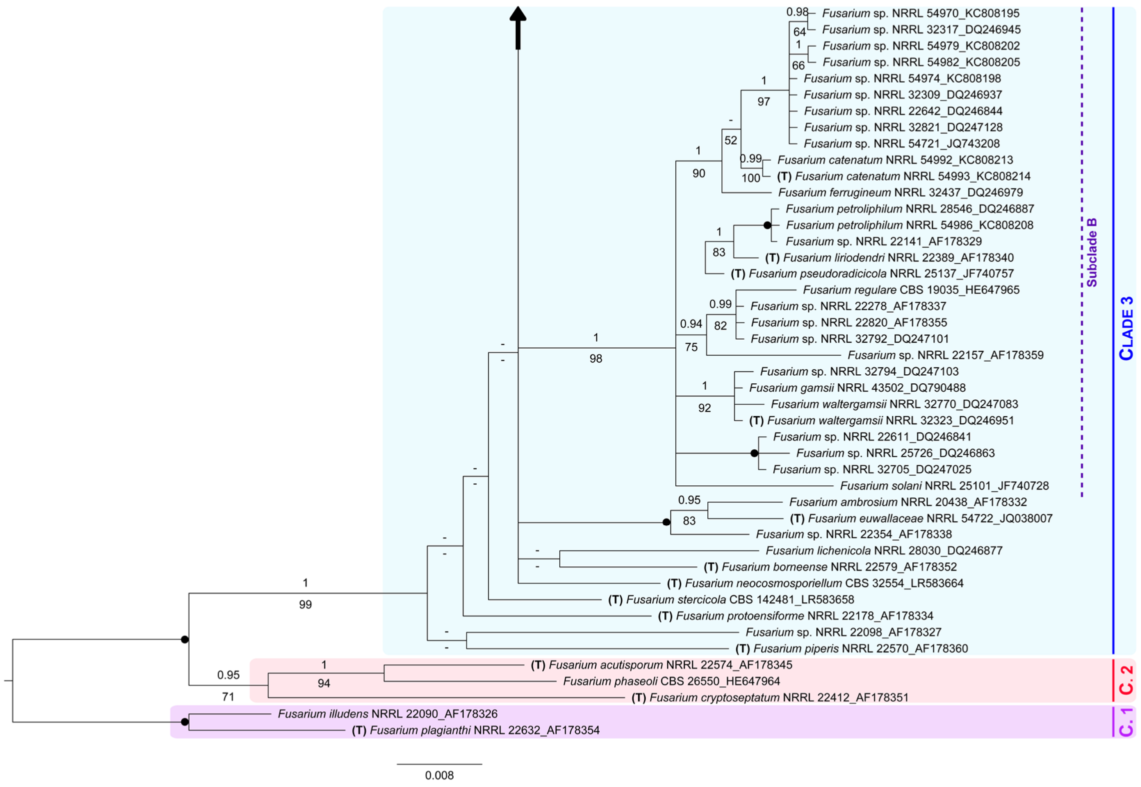

Figure S4. (Continuation).

Table S1. Fungal isolates obtained from *P. unifilis* eggshells, and putative identity based on ITS data.

Isolate	Geographic origin	Putative species name	ITS GenBank Accession No.	Identity	Seq. newly generated
143 FUS[b]	Guiyero	*Fusarium solani*	MT447548	100%	MW390926
		Fusarium keratoplasticum	MN907495	100%	
144 FUS[b]	Guiyero	*Trichoderma pubescens**	MN644760	100%	MW390927
		Undetermined fungal sp.	KY7760381	100%	
		Fusarium sp.	KU9507271	100%	
		Fusarium keratoplasticum	KP132216	100%	
145 FUS[b]	Guiyero	*Fusarium falciforme*	MT114705	100%	MW390928
		Fusarium solani	MH7290211	100%	
		Uncultured fungus clone	KF0602101	100%	
146 FUS[a]	Guiyero	*Rhizopus microsporus*	MT279280	100%	MW390929
151 FUS[b]	Guiyero	Uncultured fungus clone	MF510685	100%	MW390930
		Fusarium sp.	KX953611	100%	
		Fusarium solani	KY484924	100%	
152 FUS[b]	Nueva Providencia	*Fusarium falciforme*	MT114705	100%	MW390931
		Fusarium solani	MH729021	100%	
		Fusarium sp.	JQ364977	100%	
153 FUS[b]	Sani Isla	*Fusarium solani*	KT441029	100%	MW390932
		Fusarium sp.	EF687945	100%	
154 FUS[b]	Sani Isla	*Fusarium solani*	MT447548	100%	MW390933
		Fusarium keratoplasticum	MN907486	100%	
		Fusarium sp.	MH681596	100%	
		Uncultured fungus clone	MF510804	100%	
		Nectria haematococca[c]	KF679815	100%	
155 FUS	Sani Isla	Chaetomiaceae sp.	MT530063	100%	MW390934
		Chaetomium homopilatum	MT035919	100%	
		Humicola fuscoatra	MG774450	100%	
		Uncultured soil fungus	EU826907	100%	
156 FUS[b]	Sani Isla	*Fusarium solani*	MT447548	100%	MW390935
		Fusarium keratoplasticum	MN907486	100%	
		Fusarium sp.	MH681596	100%	
		Uncultured fungus clone	MF510804	100%	
		Nectria haematococca[c]	KF679815	100%	
157 FUS[b]	Sani Isla	*Fusarium solani*	MT447548	100%	MW390936
		Fusarium keratoplasticum	MN907486	100%	
		Fusarium sp.	MH681596	100%	
		Uncultured fungus clone	MF510804	100%	
		Nectria haematococca[c]	KF679815	100%	
158 FUS[b]	Undetermined	*Fusarium solani*	MT447548	100%	MW390937
		Fusarium keratoplasticum	MN907486	100%	
		Fusarium sp.	MH681596	100%	
		Uncultured fungus clone	MF510804	100%	
		Nectria haematococca[c]	KF679815	100%	
159 FUS	Undetermined	*Ectophoma pomi*	MT112289	99.80%	MW390938
		Phoma pereupyrena[d]	KJ767076	99.80%	
		Paraphoma radicina[e]	MH425313	99.80%	
160 FUS[b]	Undetermined	*Fusarium solani*	MT447548	100%	MW390939
		Fusarium keratoplasticum	MN907486	100%	
		Fusarium sp.	MH681596	100%	
		Uncultured fungus clone	MF510804	100%	
		Nectria haematococca	KF679815	100%	

[a]Isolates identified at species level (maximum sequence similarity with GenBank sequences representing a single species). [b]Isolates selected for phylogenetic analyses. [c]Teleomorph of *Fusarium solani*. [d]Synonym of *Ectophoma pomi*. [e]Previously named as *Phoma radicina*. [f]Teleomorph of *Fusarium verticillioides*. *Wrong identification (based on BLAST searches, all the most similar sequences to this one correspond to *Fusarium* sp.).

Table S1. (Continuation).

161 FUS[b]	Undetermined	*Fusarium solani*	MT447548	100%	MW390940
		Fusarium keratoplasticum	MN907495	100%	
		Fusarium sp.	MH681596	100%	
		Uncultured fungus clone	MF510804	100%	
		Nectria haematococca[c]	KF679815	100%	
162 FUS[b]	Undetermined	*Fusarium solani*	MT447548	100%	MW390941
		Fusarium keratoplasticum	MN907495	100%	
		Fusarium sp.	MH681596	100%	
		Uncultured fungus clone	MF510804	100%	
		Nectria haematococca	KF679815	100%	
163 FUS[b]	Undetermined	*Fusarium solani*	MT447548	100%	MW390942
		Fusarium keratoplasticum	MN907486	100%	
		Fusarium sp.	MH681596	100%	
		Uncultured fungus clone	MF510804	100%	
		Nectria haematococca	KF679815	100%	
164 FUS	Undetermined	*Penicillium expansum*	MT582774	100%	MW390943
		Penicillium crustosum	MT582770	100%	
		Penicillium commune	MN371392	100%	
		Penicillium christenseniae	MK267452	100%	
		Penicillium cyclopium	MH865559	100%	
165 FUS[a]	Undetermined	*Rhizopus microsporus*	MT279280	100%	MW390944
166 FUS[a]	Undetermined	*Rhizopus microsporus*	MT279280	100%	MW390945
168 FUS	Undetermined	*Fusarium redolens*	MT563395	100%	MW390946
		Fusarium proliferatum	MT541890	100%	
		Fusarium concentricum	MN341308	100%	
		Fusarium oxysporum	MN856435	100%	
		Fusarium solani	MN486565	100%	
		Fusarium verticillioides	MH087117	100%	
		Fusarium fujikuroi	KP998524	100%	
		Gibberella moniliformis[f]	JX914478	100%	
170 FUS	Undetermined	*Fusarium redolens*	MT563395	99.81%	MW390947
		Fusarium proliferatum	MT541890	99.81%	
		Fusarium sp.	MK351450	99.81%	
		Fusarium oxysporum	MN856435	99.81%	
		Fusarium solani	MN486565	99.81%	
		Fusarium concentricum	MH613764	99.81%	
		Fusarium verticillioides	MH087117	99.81%	
		Fusarium fujikuroi	KP998524	99.81%	
178 FUS	Undetermined	*Allophoma minor*	MH8615011	100%	MW390948
		Didymella sp.	KY828938	100%	
		Fungal species	KY4968361	100%	
197 FUS[b]	Guiyero	*Fusarium solani*	MN066126	100%	MW390949
		Fusarium sp.	MH681596	100%	
		Uncultured fungus clone	MF510804	100%	
		Fusarium keratoplasticum	KF255446	100%	
		Nectria haematococca	DQ535183	100%	
198 FUS[b]	Sani Isla	*Fusarium solani*	MN066126	100%	MW390950
		Fusarium sp.	MH681596	100%	
		Uncultured fungus clone	MF510804	100%	
		Fusarium keratoplasticum	KF255446	100%	
		Nectria haematococca	DQ535183	100%	
199 FUS	Nueva Providencia	*Fusarium oxysporum*	MT530243	100%	MW390951
		Fusarium solani	MN452609	100%	
		Trichoderma sp.*	MK871244	100%	
200 FUS[b]	Sani Isla	*Fusarium solani*	MN066126	100%	MW390952
		Fusarium sp.	MH681596	100%	
		Uncultured fungus clone	MF510804	100%	
		Fusarium keratoplasticum	KF255446	100%	
		Nectria haematococca	DQ535183	100%	
205 FUS[a]	Guiyero	*Rhizopus microsporus*	KY828894	99.85%	MW390953

Note: based on BLAST searches, the specific identity of most of these isolates could not be determined since their ITS sequences shared high similarity values with several GenBank sequences representing different species. At genus level, 20 isolates belonged to *Fusarium*, four to *Rhizopus*, and one to *Penicillium*. As for the remaining three isolates (155 FUS, 159 FUS and 178 FUS), their generic identity could not be determined as they all showed high sequence similarity with several GenBank sequences corresponding to different genera, i.e., *Chaetomium* or *Humicola*, *Ectophoma* or *Paraphoma*, and *Allophoma* or *Didymella*, respectively. Among the 20 *Fusarium* isolates, 17 (85%) were identified as species of the FSSC Clade 3. Based on BLAST results, the remaining three isolates (168 FUS, 170 FUS and 199 FUS) were tentatively assigned to species belonging to other complexes, i.e, *Fusarium fujikuroi* (FFSC), *Fusarium oxysporum* (FOSC) or *Fusarium redolens* (FRSC). All four isolates representing the genus *Rhizopus* were unequivocally identified as *Rhizopus microsporus*. The specific identity of the isolate 164 FUS remains unkwown as its sequence of ITS shared maximum similarity with GenBank sequences obtained from five *Penicillium* species. The isolate 159 FUS was neither unequivocally identified at species level since it matched with several GenBank sequences representing *Ectophoma pomi* (MT112289 and KJ767076) and *Paraphoma radicina* (MH425313).

Table S2. GenBank sequences from 130 fungal specimens used in phylogenetic analyses.

Species ID in GenBank	Isolate[a–e]	GenBank Accession No.			Host
		LSU	ITS	EF-1α	
Fusarium acutisporum	NRRL 22574*	AF178378	NR_169884	AF178345	–
Fusarium ambrosium	NRRL 20438	DQ236357	DQ094315	AF178332	Tea plant
Fusarium bataticola	NRRL 22402*	AF178377	AF178408	AF178344	*Ipomoea batatas*
Fusarium borneense	NRRL 22579*	AF178384	AF178415	AF178352	Bark
Fusarium catenatum	NRRL 54992	KC808255	KC808255	KC808213	Unknown host
Fusarium catenatum	NRRL 54993*	KC808256	KC808256	KC808214	Unknown host
Fusarium crassum	CBS 144386*	LR583709	LR583709	LR583604	–
Fusarium croci	CPC 27186*	LT746265	LT746265	LT746216	*Citrus sinensis*
Fusarium cryptoseptatum	NRRL 22412*	AF178383	AF178414	AF178351	–
Fusarium euwallaceae	NRRL 54722*	JQ038014	JQ038014	JQ038007	–
Fusarium falciforme	024 FUS	KC573882	KC573925	–	*Eretmochelys*
Fusarium falciforme	033 FUS	KC573883	KC573932	–	*Chelonia mydas*
Fusarium falciforme	NRRL 22781	DQ236376	DQ094334	DQ246849	Human
Fusarium falciforme	NRRL 22938	DQ236380	DQ094338	DQ246855	Human
Fusarium falciforme	NRRL 25456	DQ236386	DQ094344	DQ246862	Human
Fusarium falciforme	NRRL 28351	DQ236400	DQ094358	DQ246881	Human
Fusarium falciforme	NRRL 28548	DQ236405	DQ094363	DQ246889	Human
Fusarium falciforme	NRRL 28549	DQ236406	DQ094364	DQ246890	Human
Fusarium falciforme	NRRL 28556	DQ236412	DQ094370	DQ246897	Human
Fusarium falciforme	NRRL 28562	DQ236418	DQ094376	DQ246903	Human
Fusarium falciforme	NRRL 32307	DQ236447	DQ094405	DQ246935	Human
Fusarium falciforme	NRRL 32308	DQ236448	DQ094406	DQ246936	Human
Fusarium falciforme	NRRL 32312	DQ236452	DQ094410	DQ246940	Human
Fusarium falciforme	NRRL 32339	DQ236478	DQ094436	DQ246967	Human
Fusarium falciforme	NRRL 32502	DQ236507	DQ094465	DQ246999	Human
Fusarium falciforme	NRRL 32505	DQ236509	DQ094467	DQ247002	Human
Fusarium falciforme	NRRL 32538	DQ236511	DQ094469	DQ247004	Human
Fusarium falciforme	NRRL 32540	DQ236513	DQ094471	DQ247006	Human
Fusarium falciforme	NRRL 32542	DQ236515	DQ094473	DQ247008	Human
Fusarium falciforme	NRRL 32545	DQ236518	DQ094476	DQ247011	Human
Fusarium falciforme	NRRL 32714	DQ236538	DQ094496	DQ247034	Human
Fusarium falciforme	NRRL 32778	DQ236591	DQ094549	DQ247088	Horse
Fusarium falciforme	NRRL 32790	DQ236601	DQ094559	DQ247099	Human
Fusarium falciforme	NRRL 32828	DQ236636	DQ094594	DQ247135	Human
Fusarium falciforme	NRRL 32872	DQ236665	DQ094623	DQ247169	Dog
Fusarium falciforme	NRRL 32928	DQ236672	DQ094630	DQ247176	Human
Fusarium falciforme	CBS 47567*	MG189915	NR_164424	LT906669	–
Fusarium ferrugineum	NRRL 32437	DQ236488	DQ094446	DQ246979	Human
Fusarium gamsii	NRRL 43502	DQ790532	DQ790532	DQ790488	Human
Fusarium illudens	NRRL 22090	AF178362	AF178393	AF178326	–
Fusarium keratoplasticum	NRRL 54963	KC808230	KC808230	KC808190	Unknown host
Fusarium keratoplasticum	NRRL 54965	KC808232	KC808232	KC808192	Unknown host
Fusarium keratoplasticum	NRRL 54975	KC808240	KC808240	KC808199	Unknown host
Fusarium keratoplasticum	NRRL 54998	KC808258	KC808258	KC808216	Unknown host
Fusarium keratoplasticum	NRRL 54999	KC808259	KC808259	KC808217	Unknown host
Fusarium keratoplasticum	NRRL 62544	KC808261	KC808261	KC808219	Unknown host
Fusarium keratoplasticum	FRC S-2477*	JN235282	NR_130690	JN235712	Sink drain
Fusarium lichenicola	NRRL 28030	DQ236397	DQ094355	DQ246877	Human
Fusarium liriodendri	NRRL 22389*	AF178373	AF178404	AF178340	*L. tulipifera*
Fusarium neocosmosporiellum	CBS 32554*	KM231670	KM231803	LR583664	–

*Type material; [a]CBS = Westerdijk Fungal Biodiversity Institute, Utrecht (The Netherlands); [b]CPC = Culture collection of Pedro Crous, housed at CBS; [c]FRC = *Fusarium* Research Center, Pennsylvania State University (USA); [d]FUS = Real Jardín Botánico CSIC, Madrid (Spain); [e]NRRL = Agricultural Research Service, Peoria (USA). Specimens in square brackets were initially identified as *F. solani* by Sarmiento-Ramírez et al. [61], but actually correspond to *F. keratoplasticum* [57]. Dashes indicate lack of data.

Table S2. (Continuation).

Fusarium petroliphilum	NRRL 28546	DQ236403	DQ094361	DQ246887	Human
Fusarium petroliphilum	NRRL 54986	KC808250	KC808250	KC808208	Unknown host
Fusarium phaseoli	CBS 26550	MH868124	MH856617	HE647964	–
Fusarium piperis	NRRL 22570*	AF178391	AF178422	AF178360	*Piper nigrum*
Fusarium plagianthi	NRRL 22632*	AF178386	AF178417	AF178354	–
Fusarium protoensiforme	NRRL 22178*	AF178368	AF178399	AF178334	Dicot tree
Fusarium pseudoradicicola	NRRL 25137*	JF740899	JF740899	JF740757	–
Fusarium regulare	CBS 19035	MH867150	MH855640	HE647965	–
[*Fusarium solani*]	001 AFUS	JN939570	FR691753	–	*Caretta caretta*
[*Fusarium solani*]	001 CFUS	KC594706	FR691754	–	*Caretta caretta*
[*Fusarium solani*]	010 FUS	KC573904	FR691761	–	*Caretta caretta*
[*Fusarium solani*]	013 FUS	KC573907	FR691764	–	*Caretta caretta*
[*Fusarium solani*]	014 FUS	KC573908	FR691757	–	*Caretta caretta*
[*Fusarium solani*]	015 FUS	KC573909	FR691759	–	*Caretta caretta*
[*Fusarium solani*]	028 FUS	KC573914	KC573927	–	*Chelonia mydas*
[*Fusarium solani*]	031 FUS	KC573917	KC573930	–	*Chelonia mydas*
[*Fusarium solani*]	034 FUS	KC573918	KC573933	–	*Chelonia mydas*
[*Fusarium solani*]	036 FUS	KC573919	KC573935	–	*Chelonia mydas*
Fusarium solani	137 FUS	KC573895	KC573984	–	Aquarium
Fusarium solani	138 FUS	KC573896	KC573985	–	Aquarium
[*Fusarium solani*]	228 FUS	KC573921	KC574009	–	*Chelonia mydas*
Fusarium solani	NRRL 22779	DQ236375	DQ094333	DQ246848	Human
Fusarium solani	NRRL 22783	DQ236377	DQ094335	DQ246851	Seal
Fusarium solani	NRRL 25083	JF740882	JF740882	JF740714	*Musca domestica*
Fusarium solani	NRRL 25101	JF740886	JF740886	JF740728	*Boophilus* sp.
Fusarium solani	NRRL 32484	DQ236491	DQ094449	DQ246982	Human
Fusarium solani	NRRL 32737	DQ236560	DQ094518	DQ247057	Human
Fusarium solani	NRRL 32810	DQ236619	DQ094577	DQ247118	Human
Fusarium solani	NRRL 52699	JF740905	JF740905	JF740782	*Mahanarva andigena*
Fusarium solani	NRRL 52704	JF740908	JF740908	JF740786	*Tetranychus urticae*
Fusarium solani	NRRL 52778	JF740931	JF740931	JF740846	*Eurygaster* sp.
Fusarium solani	NRRL 52798	JF740936	JF740936	JF740866	*T. myopaeformis*
Fusarium solani	CBS 140079*	KT313633	NR_163531	KT313611	–
Fusarium sp.	NRRL 54974	KC808239	KC808239	KC808198	Unknown host
Fusarium sp.	NRRL 54970	KC808236	KC808236	KC808195	Unknown host
Fusarium sp.	NRRL 32309	DQ236449	DQ094407	DQ246937	Human
Fusarium sp.	NRRL 54979	KC808244	KC808244	KC808202	Unknown host
Fusarium sp.	NRRL 54982	KC808247	KC808247	KC808205	Unknown host
Fusarium sp.	NRRL 22611	DQ236368	DQ094326	DQ246841	Human
Fusarium sp.	NRRL 22098	DQ236343	DQ094301	AF178327	Cucurbit
Fusarium sp.	NRRL 22141	DQ236349	DQ094307	AF178329	Cucurbit
Fusarium sp.	NRRL 22157	DQ236348	DQ094306	AF178359	*Morus alba*
Fusarium sp.	NRRL 22163	AF178363	AF178394	AF178328	*X. piperitum*
Fusarium sp.	NRRL 22278	DQ236351	DQ094309	AF178337	*Pisum sativum*
Fusarium sp.	NRRL 22354	AF178371	AF178402	AF178338	Bark
Fusarium sp.	NRRL 22400	DQ236345	DQ094303	AF178343	*Ipomoea batatas*
Fusarium sp.	NRRL 22640	DQ236369	DQ094327	DQ246842	Human
Fusarium sp.	NRRL 22642	DQ236371	DQ094329	DQ246844	Prawn
Fusarium sp.	NRRL 22645	DQ236372	DQ094330	DQ246845	Shrimp
Fusarium sp.	NRRL 22791	DQ236379	DQ094337	DQ246853	*Iguana* sp.
Fusarium sp.	NRRL 22820	DQ236352	DQ094310	AF178355	*Glycine max*
Fusarium sp.	NRRL 25391	DQ236385	DQ094343	DQ246860	Shrimp
Fusarium sp.	NRRL 25726	DQ236387	DQ094345	DQ246863	Human
Fusarium sp.	NRRL 28014	DQ236396	DQ094354	DQ246872	Human
Fusarium sp.	NRRL 28550	DQ236407	DQ094365	DQ246891	Human
Fusarium sp.	NRRL 31165	DQ236436	DQ094394	DQ246921	Human
Fusarium sp.	NRRL 32317	DQ236456	DQ094414	DQ246945	Treefish
Fusarium sp.	NRRL 32705	DQ236530	DQ094488	DQ247025	Human

Table S2. (Continuation).

Fusarium sp.	NRRL 32711	DQ236535	DQ094493	DQ247031	Human
Fusarium sp.	NRRL 32751	DQ236573	DQ094531	DQ247070	Human
Fusarium sp.	NRRL 32780	DQ236593	DQ094551	DQ247090	Sea turtle
Fusarium sp.	NRRL 32792	DQ236603	DQ094561	DQ247101	Human
Fusarium sp.	NRRL 32794	DQ236605	DQ094563	DQ247103	Humidifier coolant
Fusarium sp.	NRRL 32821	DQ236629	DQ094587	DQ247128	Turtle egg
Fusarium sp.	NRRL 32862	DQ236663	DQ094621	DQ247167	Unknown host
Fusarium sp.	NRRL 32959	DQ236674	DQ094632	DQ247178	Human
Fusarium sp.	NRRL 43690	EF453167	EF453167	EF453015	Human
Fusarium sp.	NRRL 46596	GU170647	GU170647	GU170627	Human
Fusarium sp.	NRRL 54721	JQ743210	JQ743210	JQ743208	*Hippocampus erectus*
Fusarium stercicola	CBS 142481*	MG250480	MG250476	LR583658	–
Fusarium striatum	NRRL 22101	AF178367	AF178398	AF178333	Cotton cloth
Fusarium suttonianum	NRRL 22608	DQ236365	DQ094323	DQ246838	Human
Fusarium suttonianum	NRRL 28000	DQ236389	DQ094347	DQ246865	Human
Fusarium suttonianum	NRRL 28001	DQ236390	DQ094348	DQ246866	Human
Fusarium suttonianum	NRRL 32316	DQ236455	DQ094413	DQ246944	Human
Fusarium suttonianum	NRRL 54972	KC808238	KC808238	KC808197	Unknown host
Fusarium suttonianum	NRRL 32858*	DQ236659	DQ094617	DQ247163	Human
Fusarium tonkinense	NRRL 32755	DQ236576	DQ094534	DQ247073	Turtle
Fusarium waltergamsii	NRRL 32770	DQ236586	DQ094544	DQ247083	Human
Fusarium waltergamsii	NRRL 32323*	DQ236462	DQ094420	DQ246951	–

Table S3. Bacteria isolated from *P. unifilis* eggshells and putative identity based on 16S Blast results.

Isolate	Geographic origin	Putative species name[a]	16S GenBank Accession No.	Identity	Phylum
B13	Nueva Providencia	*Bacillus thuringiensis*	CP050183	99.93%	Firmicutes
		Bacillus proteolyticus	MT184819	99.93%	
		Bacillus wiedmannii	AP022643	99.93%	
		Bacillus cereus	CP028009	99.93%	
		Bacillus albus	CP040344	99.93%	
		Bacillus luti	CP040336	99.93%	
		Bacillus anthracis	KM888109	99.93%	
		Bacillus bombysepticus	CP007512	99.93%	
B14	Nueva Providencia	*Bacillus wiedmannii*	AP022643	98.31%	Firmicutes
		Bacillus cereus	CP028009	98.31%	
		Bacillus thuringiensis	MT598028	98.23%	
		Bacillus proteolyticus	MT184819	98.23%	
		Bacillus paranthracis	MN904873	98.23%	
B15	Nueva Providencia	*Tsukamurella* sp.	MN075319	99.86%	Actinobacteria
		Tsukamurella paurometabola	LR131273	99.79%	
		Tsukamurella inchonensis	AB907635	99.79%	
		Tsukamurella tyrosinosolvens	FJ643549	99.44%	
B16	Guiyero	*Paracoccus* sp.	AM084045	99.86%	Proteobacteria
		Paracoccus huijuniae	MN330433	99.85%	
		Paracoccus aminovorans	HQ005404	99.78%	
B17	Guiyero	*Pimelobacter* sp.	KC252718	99.12%	Actinobacteria
		Pimelobacter simplex	NR_119355	99.09%	
		Nocardioides aromaticivorans	GU735484	99.02%	
B18	Sani Isla	*Nocardioides kongjuensis*	NR_043651	99.72%	Actinobacteria
		Nocardioides panaciterrae	AB257719	99.45%	
B19	Sani Isla	*Gordonia* sp.	KC211011	99.93%	Actinobacteria
		Uncultured actinobacterium	JQ919182	99.93%	
		Gordonia sihwensis	NR_025505	99.86%	
		Gordonia cholesterolivorans	NR_044445	99.86%	
B20	Sani Isla	*Ralstonia* sp.	AY860250	99.86%	Proteobacteria
		Cupriavidus sp.	MG725957	99.79%	
		Cupriavidus pauculus	CP044065	99.18%	
		Cupriavidus plantarum	HQ438088	99.18%	
		Ralstonia eutropha	AF027407	99.11%	
B21	Sani Isla	*Pseudoxanthomonas* sp.	LC481374	100%	Proteobacteria
		Uncultured bacterium clone	HQ218747	100%	
		Pseudoxanthomonas indica	JQ659945	100%	
		Pseudoxanthomonas japonensis	LC015564	100%	
B22	Sani Isla	*Pseudomonas* sp.	KP986946	100%	Proteobacteria
		Pseudomonas mendocina	EF208965	99.93%	
		Pseudomonas resinovorans	MF943158	99.86%	
		Pseudomonas aeruginosa	KF929419	99.79%	
B23	Sani Isla	*Pseudomonas* sp.	CP045118	99.45%	Proteobacteria
		Pseudomonas denitrificans	CP043626	99.38%	
		Pseudomonas nitroreducens	MH675504	99.38%	
		Pseudomonas multiresinivorans	CP048833	99.31%	
		Pseudomonas azelaica	AM088475	99.25%	
		Pseudomonas aeruginosa	MF144534	99.18%	
B24	Sani Isla	*Delftia* sp.	MT101746	99.93%	Proteobacteria
		Delftia tsuruhatensis	MH478206	99.93%	
		Delftia lacustris	MH333093	99.93%	
		Uncultured bacterium clone	KX508964	99.93%	
		Uncultured Comamonadaceae	JN038272	99.93%	
		Delftia acidovorans	GU459215	99.86%	
B25	Undetermined	*Chryseobacterium gleum*	KX579964	99.44%	Bacteroidetes
		Chryseobacterium aquifrigidense	KP893287	99.42%	
		Chryseobacterium flavum	MK116543	99.37%	
		Chryseobacterium indologenes	KX228235	99.37%	
		Chryseobacterium arthrosphaerae	LC040953	99.16%	

[a]None of the isolates could be ascribed to a given species since multiple hits with identical or very similar sequence identity values were found.

Table S3. (Continuation).

B26	Undetermined	*Achromobacter* sp.	LC093432	99.79%	Proteobacteria
		Uncultured bacterium clone	KR233647	99.66%	
		Achromobacter aegrifaciens	NR_117707	99.66%	
		Achromobacter xylosoxidans	CP061008	99.59%	
		Achromobacter insolitus	CP038034	99.45%	
		Achromobacter denitrificans	CP053986	99.45%	
		Achromobacter insuavis	NR_117706	99.38%	
		Achromobacter ruhlandii	CP017433	99.31%	
B27	Undetermined	*Elizabethkingia* sp.	MN540121	100%	Bacteroidetes
		Elizabethkingia anophelis	CP034594	100%	
		Elizabethkingia endophytica	CP016372	100%	
		Chryseobacterium meningosepticum	AY683476	100%	
B28	Undetermined	*Stenotrophomonas maltophilia*	LC066105	99.86%	Proteobacteria
		Stenotrophomonas sp.	MN714633	99.86%	
		Uncultured bacterium clone	EF509504	99.80%	
		Pseudomonas sp.	LC034239	99.73%	
		Pseudomonas hibiscicola	KC172017	99.66%	
		Pseudomonas geniculata	KJ147059	99.59%	
B29	Undetermined	Bacterium enrichment culture	HQ440208	99.93%	Actino- or Proteobacteria
		Pseudomonas sp.	KU291441	99.86%	
		Arthrobacter sp.	AY641537	99.79%	
		Pseudomonas plecoglossicida	KU597529	99.73%	
		Pseudomonas putida	CP015202	99.72%	
		Pseudomonas monteilii	KF475842	99.66%	
B30	Undetermined	*Stenotrophomonas* sp.	KY672922	100%	Proteobacteria
		Uncultured bacterium clone	KU667239	100%	
		Stenotrophomonas acidaminiphila	KF279369	100%	
		Stenotrophomonas maltophilia	MK562366	100%	
B31	Undetermined	*Delftia tsuruhatensis*	DQ864991	99.86%	Proteobacteria
		Delftia sp.	MT101746	99.86%	
		Delftia lacustris	MH333093	99.86%	
		Uncultured bacterium clone	KX508964	99.86%	
		Delftia acidovorans	GU459215	99.79%	
B32	Undetermined	*Pseudomonas* sp.	MH703466	99.93%	Proteobacteria
		Stenotrophomonas sp.	CP029759	99.86%	
		Stenotrophomonas maltophilia	MK841317	99.86%	
		Uncultured marine bacterium	JF925027	99.86%	
		Stenotrophomonas pavanii	MN686363	99.79%	
		[*Pseudomonas*] *geniculata*	KT748641	99.73%	
B33	Undetermined	*Stenotrophomonas maltophilia*	AB683956	99.86%	Proteobacteria
		Uncultured *Stenotrophomonas* sp.	HQ595218	99.86%	
		Pseudomonas sp.	KR911802	99.79%	
		Uncultured bacterium clone	EU805183	99.65%	
		Vibrio sp.	DQ146981	99.65%	
B34	Undetermined	*Pseudomonas putida*	AY686638	99.79%	Actino- or Proteobacteria
		Pseudomonas taiwanensis	NR_116172	99.72%	
		Pseudomonas sp.	MT012082	99.72%	
		Pseudomonas plecoglossicida	LC507998	99.59%	
		Pseudomonas monteilii	MF045811	99.59%	
		Brevibacterium frigoritolerans	EU073968	99.52%	

Note: the 22 bacterial isolates obtained here represent four different phyla, i.e., Proteobacteria (12 isolates, 54.5%), Actinobacteria (four, 18.2%), Firmicutes (two, 9.1%) and Bacteroidetes (two, 9.1%). Based on the closest matches of BLAST searches, the remaining two isolates (B29 and B34) could belong to either Actinobacteria or Proteobacteria. Among the Proteobacteria, two isolates (16.7%) were assigned to *Pseudomonas*, two others (16.7%) to *Delftia*, and B16, B21, B26 and B30 (33.3%) most probably represented *Paracoccus*, *Pseudoxanthomonas*, *Achromobacter*, and *Stenotrophomonas*, respectively. The genus of the isolates B20, B28, B32 and B33 (33.3%) could not be determined since BLAST revealed significant similarity (> 99%) to several sequences corresponding to different genera, i.e., *Ralstonia* or *Cupriavidus*, *Stenotrophomonas* or *Pseudomonas*, and *Stenotrophomonas*, *Pseudomonas* or *Vibrio*. Among the Actinobacteria, B15 corresponded to *Tsukamurella*, B18 to *Nocardioides*, B19 to *Gordonia*, and B17 could belong either to *Pimelobacter* or *Nocardioides.* Both representatives of Firmicutes (B13 and B14) corresponded to *Bacillus.* The isolates representing the phylum Bacteroidetes corresponded to *Cryseobacterium* (B25) and *Elizabethkingia* or *Cryseobacterium* (B27).

Table S4. GenBank 16S sequences from related bacterial species used in phylogenetic analyses.

Species ID in GenBank	Isolate	16S GenBank Accession No.	Fragment*
Achromobacter aegrifaciens	LMG 26852	CADIJY010000029	2–1435
Achromobacter agilis	LMG 3411	UFQB01000054	247–1773
Achromobacter animicus	R-46662T	HE613448	–
Achromobacter animicus	R-46662	NR_117615	–
Achromobacter animicus	LMG 26690	CADIJM010000037	2–1435
Achromobacter anxifer	LMG 26857	CAHLAR010000027	2–1435
Achromobacter denitrificans	DSM 30026	NR_042021	–
Achromobacter denitrificans	LMG 1231	CADIJN010000119	237–1763
Achromobacter insolitus	LCu2	CP038034	1780713–1782263
Achromobacter insolitus	LMG 6003	CADIJK010000027	45–1571
Achromobacter kerstersii	LMG 3441	CADIJQ010000032	25–1551
Achromobacter marplatensis	R-46660T	HE613447	–
Achromobacter marplatensis	R-46660	NR_117614	–
Achromobacter marplatensis	LMG 26219	CADIJU010000049	367–1893
Achromobacter marplatensis	B2	NJIG01000044	245–1690
Achromobacter mucicolens	R-46658T	HE613446	–
Achromobacter mucicolens	R-46658	NR_117613	–
Achromobacter mucicolens	LMG 26685	CADIJP010000044	163–1689
Achromobacter panacis	–	KR338991	–
Achromobacter panacis	DCY105	NR_165687	–
Achromobacter pestifer	LMG 3431	CADIJX010000020	263–1789
Achromobacter piechaudii	NBRC102461	NZ_BCTK01000022	–
Achromobacter piechaudii	LMG 1873	CADIJS010000009	10–1536
Achromobacter ruhlandii	SCCH3:ACH 33- 1365	CP017433	688341–689894
Achromobacter sp.	DR405	LC093432	–
Achromobacter sp.	Fo40	MF155647	–
Achromobacter veterisilvae	LMG 30378	UFQC01000090	50–1576
Achromobacter xylosoxidans (T)	0119Ts	MT664080	–
Achromobacter xylosoxidans (T)	GD03	CP061008	1800478-1802131
Arthrobacter sp.	LFTou2	AY641537	–
Bacillus anthracis	IHBB18033	KM888109	–
Bacillus bombysepticus	Wang	CP007512	89830–91390
Bacillus cereus	SGAir0260	CP028009	1288835–1290389
Bacillus circulans	Sn25-040808	FJ155345	–
Bacillus licheniformis	Sn13-040808	FJ155337	–
Bacillus proteolyticus	NWPZ7	MT184819	–
Bacillus pumilus	Sn6-040808	FJ155331	–
Bacillus sp.	JY14	EU581833	–
Bacillus sp.	0919U	MT664082	–
Bacillus sp.	0519Pg	MT664083	–
Bacillus subtilis (T)	Sn12-040808	FJ155336	–
Bacillus thuringiensis	HER1410	CP050183	9160–10714
Bacillus wiedmannii	PL1	AP022643	2238988–2240538
Bacterium enrichment	CCZU12	HQ440208	–
Brevibacterium frigoritolerans	CMGS4	EU073968	–
Chryseobacterium aquifrigidense	R-21	KP893287	–
Chryseobacterium arthrosphaerae	GS-3	LC040953	–
Chryseobacterium culicis	0819Tu	MT664085	–
Chryseobacterium gleum (T)	EY1	KX579964	–
Chryseobacterium hispanicum	KCTC 22104	QNUG01000096	111–1635
Chryseobacterium indologenes	Amic_5	KX228235	–
Chryseobacterium massiliae	CUVET1223	KJ190179	–
Chryseobacterium sp.	CUVET1222	KJ190178	–
Chryseobacterium sp.	D136	KF322149	–
Cupriavidus necator (T)	JS705	AF027407	–

*16S sequence obtained from a complete genome. The corresponding nucleotide range appears in the last column. T= type of the genus.

Table S4. (Continuation).

Cupriavidus pauculus	FDAARGOS	CP044065	3476779–3478401
Cupriavidus plantarum	MA1-4a	HQ438088	–
Delftia acidovorans (T)	QS1	GU459215	–
Delftia tsuruhatensis	–	DQ864991	–
Elizabethkingia anophelis	JM-87 15-247	MAGY01000034	251–1772
Elizabethkingia endophytica	JM87	CP016372	1224828–1226361
Elizabethkingia meningoseptica (T)	YB-29	AY683476	–
Gordonia shandongensis	DSM45094	NZ_AUHE01000029	–
Gordonia sp.	LYS13	KC211011	–
Mesobacillus campisalis	SA2-6	NZ_LAYY01000064	1515–2053
Nocardioides agariphilus	KCTC 19276	NZ_JADKPO01000007	351–1891
Nocardioides aromaticivorans	SB10005	GU735484	–
Nocardioides panaciterrae	KCTC 19136	AB257719	–
Nocardioides szechwanensis	CGMCC 1.11147	FNIC01000016	369–1908
Paracoccus aminovorans	CT	HQ005404	–
Paracoccus sp.	C84	KF322148	–
Paracoccus sp.	R24650	AM084045	–
Pimelobacter sp.	N044	KC252718	–
Pseudomonas aeruginosa (T)	VITLWS3	KF929419	–
Pseudomonas denitrificans	BG1	CP043626	676315–677857
Pseudomonas filiscindens	ATCC BAA-697	AY259924	–
Pseudomonas geniculata	OTU-a9	KJ147059	–
Pseudomonas geniculata	C8	KT748641	–
Pseudomonas hibiscicola	SH8	KC172017	–
Pseudomonas mendocina	B6-1	EF208965	–
Pseudomonas monteilii	IHB B 2329	KF475842	–
Pseudomonas multiresinivorans	populi	CP048833	817172–818740
Pseudomonas nitroreducens	DSM 9128	AM088475	–
Pseudomonas plecoglossicida	RD_AZPVI_05	KU597529	–
Pseudomonas plecoglossicida	JCM 13970	LC507998	–
Pseudomonas putida	KL33	AY686638	–
Pseudomonas putida	B62	CP015202	374165–375716
Pseudomonas sp.	Fenol2C	KU291441	–
Pseudomonas sp.	LS3K	LC034239	–
Pseudomonas sp.	R5	KR911802	–
Pseudomonas sp.	T2	GU727815	–
Pseudomonas sp.	PVR-YHB-1-2	KP986946	–
Pseudomonas sp.	PT1	MK652307	–
Pseudomonas sp.	PT2	MK652308	–
Pseudomonas sp.	PT4	MK652310	–
Pseudomonas sp.	PT5	MK652311	–
Pseudomonas sp.	PT6	MK652312	–
Pseudomonas sp.	PT7	MK652313	–
Pseudomonas sp.	J1	MN128405	–
Pseudomonas sp.	0619Th	MT664098	–
Pseudoxanthomonas indica	R8-542	JQ659945	–
Pseudoxanthomonas japonensis	AF59	LC015564	–
Pseudoxanthomonas jiangsuensis	DSM 22398	PDWL01000056	388–1932
Pseudoxanthomonas sp.	45-43	LC481374	–
Ralstonia sp.	AU5980	AY860250	–
Stenotrophomonas acidaminiphila	SR50-5	KF279369	–
Stenotrophomonas maltophilia (T)	TI-1	AB683956	–
Stenotrophomonas maltophilia (T)	T6220-6-1b	LC066105	–
Stenotrophomonas rhizophila	0919Es	MT664102	–
Stenotrophomonas sp.	BAB-6435	KY672922	–
Stenotrophomonas sp.	pho	CP029759	351352–352892
Tsukamurella inchonensis	1325	AB907635	–
Tsukamurella pulmonis	Sn10-040808	FJ155334	–

Table S4. (Continuation).

Tsukamurella tyrosinosolvens	Bx-62986	FJ643549	–
Uncultured *Achromobacter* sp.	ME128	DQ917890	–
Uncultured actinobacterium	GC0AA1ZA07PP1	JQ919182	–
Uncultured bacterium	P7D82-599	EF509504	–
Uncultured bacterium	6C233176	EU805183	–
Uncultured bacterium	CN1-94	HQ218747	–
Uncultured bacterium	15203	KR233647	–
Uncultured bacterium	Ace13(9-10)	KU667239	–
Uncultured bacterium	MTDH201308-62	KX508964	–
Uncultured Comamonadaceae	EK_An354	JN038272	–
Uncultured marine bacterium	Tc-42	JF925027	–
Uncultured *Stenotrophomonas* sp.	IC3081	HQ595218	–
Vibrio atlanticus	Evh13	NZ_OSDW01000040	408–1957
Vibrio cincinnatiensis	DSM19608	FUXB01000057	–
Vibrio parahaemolyticus	CFSAN025059	LKQH01000018	–
Vibrio sp.	S1072	FJ457349	–
Vibrio sp.	S1073	FJ457350	–
Vibrio sp.	S1099	FJ457358	–
Vibrio sp.	S1100	FJ457359	–
Vibrio sp.	S1101	FJ457360	–
Vibrio sp.	S1104	FJ457361	–
Vibrio sp.	S1105	FJ457362	–
Vibrio sp.	V205	DQ146981	–
Vibrio viridaestus	LJC006	RJVQ01000027	418–1907
